# Two-dimensional halide perovskite as β-ray scintillator for nuclear radiation monitoring

**DOI:** 10.1038/s41467-020-17114-7

**Published:** 2020-07-07

**Authors:** Dejian Yu, Peng Wang, Fei Cao, Yu Gu, Jiaxin Liu, Zeyao Han, Bo Huang, Yousheng Zou, Xiaobao Xu, Haibo Zeng

**Affiliations:** 1 MIIT Key Laboratory of Advanced Display Materials and Devices, Institute of Optoelectronics & Nanomaterials, College of Materials Science and Engineering, Nanjing University of Science and Technology, 210094 Nanjing, China; 2 School of Environmental and Biological Engineering, Nanjing University of Science and Technology, 210094 Nanjing, China

**Keywords:** Materials science, Nanoscience and technology

## Abstract

Ensuring nuclear safety has become of great significance as nuclear power is playing an increasingly important role in supplying worldwide electricity. β-ray monitoring is a crucial method, but commercial organic scintillators for β-ray detection suffer from high temperature failure and irradiation damage. Here, we report a type of β-ray scintillator with good thermotolerance and irradiation hardness based on a two-dimensional halide perovskite. Comprehensive composition engineering and doping are carried out with the rationale elaborated. Consequently, effective β-ray scintillation is obtained, the scintillator shows satisfactory thermal quenching and high decomposition temperature, no functionality decay or hysteresis is observed after an accumulated radiation dose of 10 kGy (dose rate 0.67 kGy h^−1^). Besides, the two-dimensional halide perovskite β-ray scintillator also overcomes the notorious intrinsic water instability, and benefits from low-cost aqueous synthesis along with superior waterproofness, thus paving the way towards practical application.

## Abstract (web summary)

Efficient radiation monitoring ensures safety in nuclear power, but beta-ray scintillators should be developed for use near a highly radioactive and hot reactor. Here, the authors report a two-dimensional halide perovskite-based beta-ray scintillator with high irradiation hardness and thermotolerance.

## Introduction

Clean energy is currently attracting more and more worldwide attention due to growing global environmental problems, including greenhouse effects and ocean acidification^1–3^, that stem from the heavy reliance on fossil fuels. While typical clean energies such as hydropower, solar energy, and wind power still face issues of geographic restriction and long payback period, nuclear energy has rapidly developed as one of the most important electricity suppliers on earth since the 1940s^4^. According to the latest world nuclear industry status report (2013), 434 nuclear power plants had been built in 30 countries, and produced 371.7 GW of electric power in 2013. Statistics of the World Bank and the International Atomic Energy Agency (IAEA) pointed out that the proportion of global nuclear power generation had increased from 2.00% in 1971 to 12% in 2016, making it the third largest source of electricity in the world (after hydropower and fossil fuels), and the proportion is still increasing. With the constant depletion of fossil fuels^5^, nuclear energy becomes increasingly important for a long time to come. However, accidents in Chernobyl and Fukushima serve as poignant reminders that disasters may occur if nuclear leakage is not well prevented, developing accurate and efficient radiation monitoring technologies is an important means to ensure the safe development of nuclear energy.

Nuclear reaction mainly releases four types of high-energy rays: α-ray, β-ray, γ-ray, and neutron ray. Among them, β-ray with a moderate penetrating power is an important signal for surface radiative contamination surveillance^6,7^. In general, scintillator-based detectors that convert high-energy rays into visible light are facile, efficient, and cost-effective, hence are widely used for radiation detection. However, although the commercial inorganic scintillators for X/γ rays are quite mature, they cannot be directly applied to β-ray sensing owing to the fundamentally different material requirements. In principle, high-*Z* (*Z* is atomic number) elements are needed for X/γ-ray scintillators for enhanced absorbtion^8^, exactly the other way around, light elements are favored in β-ray scintillator^9,10^. One important consideration is that the heavy elements lower the capturing efficiency of β particles and cause unfavorable energy allocation to fluorescence response. Besides, the low *Z* in scintillator can also suppress the background interference signal from X/γ-rays.

Currently, organic scintillators (including single-crystal, liquid, and plastic types) are mainly used for β-ray detection. However, organic scintillators are plagued by the issues of carcinogenicity, high cost, complex fabrication, poor irradiation hardness, or thermal deterioration. For example, the difficulties of crystal growth in organic single-crystal scintillators result in high cost and low availability of large, high-quality single crystals^11,12^. Even worse, being subject to sublimation, the organic single-crystal scintillators are also carcinogenic, such as anthracene and naphthalene. For liquid scintillators, they are always composed of flammable oil, the flash points are very low, therefore careful encapsulation and placement away from heat sources are required^13^. Liquid scintillators are also highly sensitive to oxygen, which imposes great challenges on fabrication, transport, and storage^14^; Compared with the single-crystal type and liquid type organic scintillators, plastic scintillators are the mainstream. They are composed of base materials, a primary fluor and a second fluor. The base materials firstly absorb the β-ray energy, and then transfer the energy to the primary fluor and subsequently to the second fluor. The problem is that the widely used bases of polystyrene (PS), polymethyl methacrylate (PMMA), and polyvinyltoluene (PVT) are restricted by a low glass transition temperature of <105 °C^15,16^, and they suffer from poor irradiation hardness^17^. All these limitations urge for the development of innovative scintillators that overcome these problems.

In the past decade, organic–inorganic hybrid metal halide perovskites (HPs) emerged as a family of unique semiconductor featuring compositional versatility and superior optical properties, such as high quantum yield (QY), adjustable bandgap, and high color purity^18^. They have quickly gained stunning progress in optoelectronic applications, including solar cells^19^, light emitting diodes^20^, photodetectors^21^, etc. However, the poor stability toward moisture, light, and heat has greatly hindered their real-life use. Recently, several groups proposed that HPs can be applied for efficient X-ray and γ-ray sensing in the form of either semiconductors or scintillators^22–37^. Further study also confirms that HPs are quite stable toward these high-energy rays^22–27,38^, making ionizing radiation detection a killer grade application of HPs. Significantly, the superior optoelectronic properties also open the possibility of β-ray detection application with HPs, but they suffer from poor stability against charged beams, whether or not and how HPs can be engineered for β-ray detection remain elusive.

In this work, a type of β-ray scintillator based on two-dimensional (2D) HPs is demonstrated. Comprehensive compositional engineering was carried out towards β-ray sensing: (1) a series of bulky organic cations in 2D HPs were explored to enhance the capturing of β particles, therefore significantly increasing the scintillation response to β-ray; (2) Extrinsic manganese (Mn(II)) dopants were incorporated, which not only serve as extra emitting centers, but also play as emission shifter to eliminate the self-absorption caused photon loss. Consequently, the 2D HP scintillators demonstrate superior irradiation hardness and heat robustness, coupled with efficient scintillation response, low-detection limit and weak afterglow. Moreover, the 2D HP scintillators also afford aqueous synthesis and waterproofness that cannot be reached by typical HP materials. We believe this work will extend the application of HPs to advance the β-ray detection technology for nuclear radiation monitoring.

## Results

### The effect of light element

**Fig. 1:**
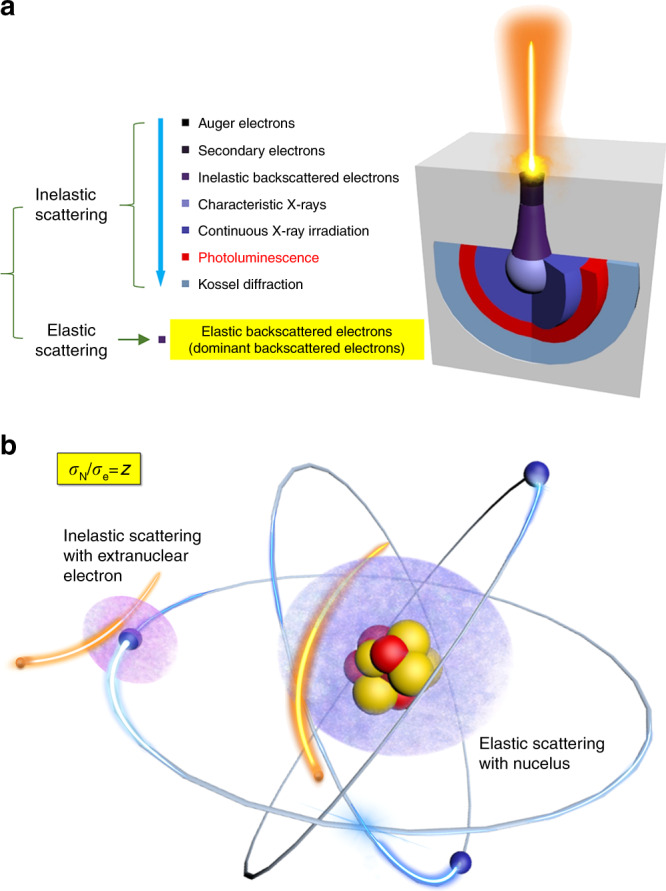
Interactions between β-ray and solids. **a** Schematic illustration of sequential signals produced by the interactions between β particles and solids via inelastic scattering. **b** Schematic view of elastic scattering with nucleus and inelastic scattering with extranuclear electrons for incident β particles. *A*_N_ and *A*_e_ represent the cross sections of the elastic scattering with nucleus and inelastic scattering with extranuclear electrons.

**Fig. 2:**
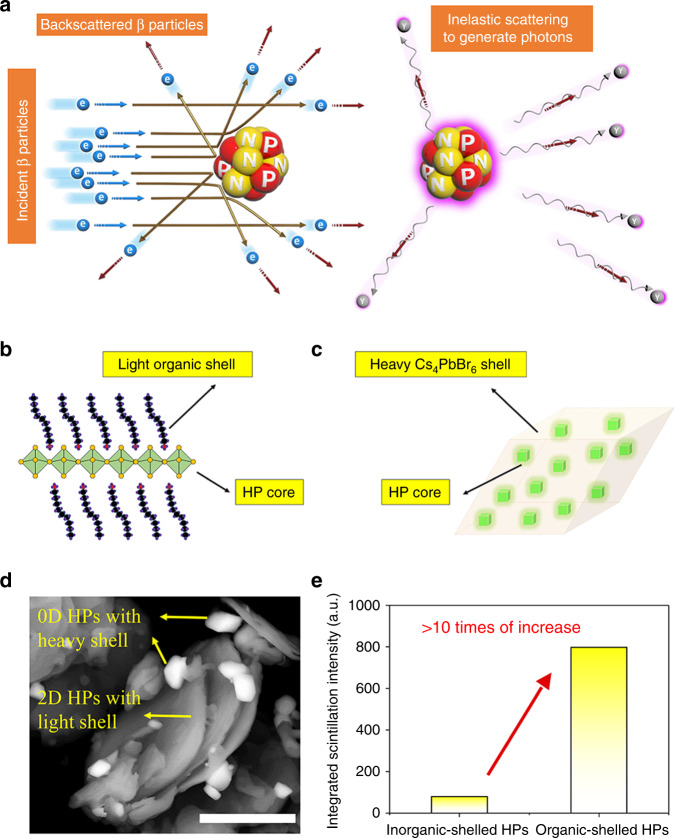
Composition-dependent β particle capturing efficiency. **a** Schematic presentation of the scattering events of incident β particles. For simplicity, extranuclear electrons are omitted from the model. Schematic views of **b** HPs with light organic shells (DA_2_PbBr_4_) and **c** HPs with heavy inorganic shells (CsPbBr_3_@Cs_4_PbBr_6_). **d** SEM image of the mixture of the two samples in backscattering electron mode, the scale bar is 5 μm. **e** The scintillation intensity comparison between the two samples.

It will be useful to present a simplified view of how β-ray interacts with scintillators. The incident β particles go through elastic scattering with nuclei and inelastic scattering with electrons in solids^28–30^. While the elastic scattering, causing dominant backscattered electrons, only changes the trajectory of β particles but imposes no change on the kinetic energy, the inelastic scattering process otherwise transfers the power of β particles to the solids to produce multiple kinds of signals in sequence (Fig. 1a), including electronic signals (auger electrons, secondary electrons, and minor inelastic backscattered electrons) and photonic signals (characteristic X-ray, continuous X-ray ray, and fluorescence). Figure 1b provides the schematic diagram of the scattering model in single atom, the intensity ratio of elastic scattering to inelastic scattering is *Z* (see details in the Methods section). This sheds light on the experience that low-*Z* elements rather than high-*Z* elements are favored in β-ray scintillators^31^, that is, light elements increase the capturing efficiency of β particles, while high-*Z* elements cause a significant escape of backscattered β particles owing to intensified elastic scattering (Fig. 2a). The value was reported to reach even up to 80–90% in typical heavy inorganic scintillators^32^. Usually, the fraction of incident β particles that reappear as BSE (known as backscattered scattering electron coefficient *η*) can be determined by the following equation^33^:

1 $$\eta = {\mathrm{ln}}Z/6 - 1/4\quad (Z \ge 10)$$

And for compounds with complex compositions, the Eq. 1 is amended as:

2 $$\bar \eta = \mathop {\sum}\limits_{i = 1}^n {C_i\eta _i}$$

where *C*_*i*_ is weight concentration of each element, *η*_*i*_ is the BSE coefficient of each element, *n* is the number of elements in the compound. In this regard, incorporation of light elements is necessary when applying HP scintillators for β-ray detection.

Another drawback that limits HP materials is the poor sensitivity toward charged beams. Milosavljevic et al.^34^ reported accelerated degradation of HP film upon increasing electron beam energy even in a quite low range from 4.5 eV to 60 eV. Dang et al.^35^ found that high-energy electron irradiation stimulates desorption of partial halogen atoms in HP nanocrystals and reduces Pb^2+^ to Pb^0^, which then aggregates to be a high-contrast particle. The same phenomenon has also been observed in other groups^36,37,39–41^. Manna’s group found that even at a low temperature (<40 °C), the damage of HP nanocrystals under the exposure of typical transmission electron microscope (TEM, 80/200 keV) is inevitable^42^. Therefore, the issue of poor irradiation robustness should be addressed before HP-type scintillators are applied for β-ray detection.

In light of the above considerations, 2D HPs (Fig. 2b) seem to be a compelling candidate, the organic components are incorporated to strengthen the lattice^43^, therefore improving the β-ray irradiation hardness. More importantly, the organic components are comprised of light elements including C (*Z* = 6), H (*Z* = 1), and N (*Z* = 7), which lower the $\bar Z$ value compared with 3D HPs to increase the capturing efficiency of β-ray. To evaluate the effect of composition on the scintillation response toward β-ray, the so-called “zero-dimensional” (0D) HP CsPbBr_3_@Cs_4_PbBr_6_ is used for reference^18,44^. The 0D HP has Cs_4_PbBr_6_ serving as the protection shell against β-ray damage (Fig. 2c), which is on the contrary composed of heavy Cs (*Z* = 55), Pb (*Z* = 82), and Br (*Z* = 35). Here DA_2_PbBr_4_ (C_24_H_54_N_2_PbBr_4_, DA is dodecylamine) is selected as the representative 2D HP, a doping strategy is applied to improve the PL QY of the 2D HP as will be introduced in the following. The two samples are fabricated both in the form of powder with close photoluminescence (PL) QY (~60% for CsPbBr_3_@Cs_4_PbBr_6_ and ~55% for 2D HP) to ensure comparability. The β particle BSE coefficients of the two systems are then evaluated according to Eqs. 1 and 2. The $\bar \eta$ of 0D HP is calculated to be 39.96%, which means a quite large portion of incident particles are backscattered from the CsPbBr_3_@Cs_4_PbBr_6_ surface. Such scattering waste in DA_2_PbBr_4_ is largely meliorated owing to the incorporation of light elements, the $\bar \eta$ value is calculated to be 24.59%, nearly half of that of the 0D counterpart. For experimental verification of the reduction in backscattering waste, scanning electron microscopy (SEM) in backscattered electron mode was carried out, in which case the backscattered electrons from samples were detected, and the contrast implies the variation in the intensity. The image is shown in Fig. 2d. Indeed, it can be clearly observed that dark 2D layered DA_2_PbBr_4_ are mixed with bright CsPbBr_3_@Cs_4_PbBr_6_ particles (characterization is in Supplementary Fig. 1) with a parallelepiped-like shape^45^. The backscattered coefficients of a series of commercial inorganic scintillators are listed and compared in Table 1.

**Table 1:** Comparison between the ${\bar{\eta}}$ value of 2D HP scintillator and that of several typical scintillators.

Scintillator	BaF_2_	NaI	LaBr_3_	CsI	DA_2_PbBr_4_
${\bar{\eta}}$	35.14%	37.15%	37.24%	41.49%	24.59%

After β particles are absorbed by 2D HPs, the incorporation of light elements also enhances the scintillation response by suppressing energy allocation on useless signals. Specifically, the photonic signals by β-ray excitation include high-energy X-ray and low-energy fluorescence (the scintillation response). Bremsstrahlung effect is the cause of continuous X-ray signal, i.e., the energy of β particle is consumed by releasing continuous X rays when high-speed charges suddenly accelerate or decelerate. The intensity of bremsstrahlung is proportional to *Z*^2^, which means a weakened bremsstrahlung effect with light elements. The $\bar Z$ of compounds is given by Eq. 3:

3 $$\bar Z = {\sum} {\left( {NZA} \right)} /{\sum} {\left( {NA} \right)}$$

where *N* is the number of atoms of each element with atomic weight *A* and atomic number *Z*. The $\bar Z$ values of 0D and 2D HP systems are calculated to be 43 and 33.6, which indicates that 39% energy consumption of high-energy X ray will be reduced to compensate the competing low-energy fluorescence signal. As a consequence of the strengthened β-ray capture and improved energy allocation, the scintillation response of 2D HP is >10 times larger than that of the 0D analog (Fig. 2e) when subject to β-ray excitation (^63^Ni, activity: 25 mCi). Corresponding scintillation spectra can be found in Supplementary Fig. 2.

**Fig. 3:**
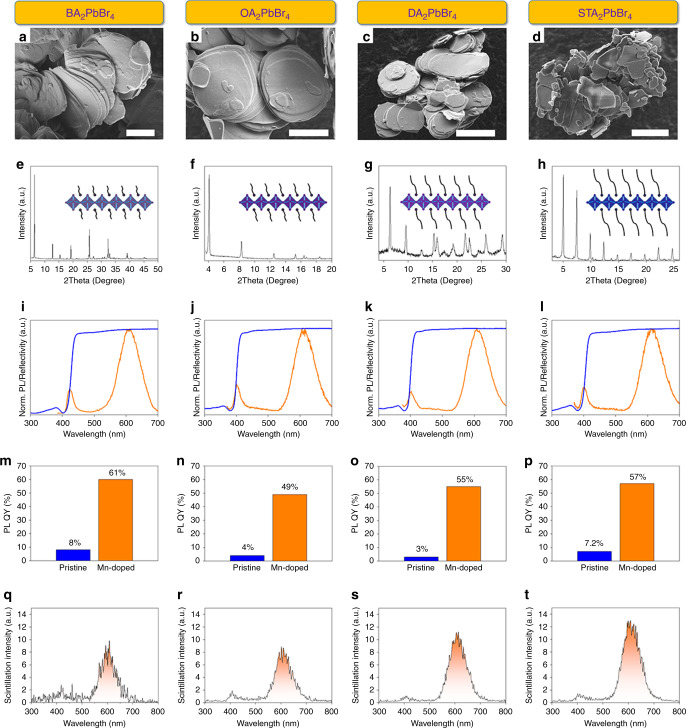
Composition-dependent β-ray scintillation. **a**–**d** SEM images of as-prepared BA_2_PbBr_4_, OA_2_PbBr_4_, DA_2_PbBr_4_, and STA_2_PbBr_4_, respectively. Corresponding scale bars are 5 μm, 5 μm, 10  μm, and 5 μm. **e**–**h** XRD patterns of BA_2_PbBr_4_, OA_2_PbBr_4_, DA_2_PbBr_4_, and STA_2_PbBr_4_, respectively. The insets are the schematic views of corresponding structures. **i**–**l** Normalized PL and reflectivity spectra of the 2D HPs with Mn(II) dopants. **m**–**p** The increase of PL QYs after Mn(II) incorporation in the 2D HPs. **q**–**t** Comparison between the scintillation performances in the 2D HPs. Source data are provided as a Source Data file.

Further, a series of 2D HPs with various organic cations (butylamine (BA), octylamine (OA), and stearamine (STA) in addition to DA) were fabricated. Corresponding SEM images are showcased in Fig. 3a–d. Following a 2D crystallographic structure, all the four kinds of 2D HPs present an obvious layered morphology. The X-ray diffraction (XRD) patterns (Fig. 3e–h) show periodic diffraction peaks in the small 2*θ* range owing to a large interlayer spacing in 2D HPs along the [00*l*] direction^46^. The interlayer spacing can be calculated according to the Bragg diffraction equation, the results are 1.38 nm, 2.12 nm, 2.77 nm, and 3.60 nm in BA_2_PbBr_4_, OA_2_PbBr_4_, DA_2_PbBr_4_, and STA_2_PbBr_4_, respectively. The large interlayer spacing greatly reduces the interactions between adjacent [PbBr_6_]^4−^ functional layers, leading to strong exciton binding for radiative recombination^47^. However, it was found that the PL QYs of pure 2D HPs are quite low (<8%), the low PL QYs are probably caused by a strong exciton-phonon interaction in 2D HPs according to a previous report by Gong et al.^48^. Therefore, additional modification is required to improve the scintillation behavior.

Here, Mn(II) dopants as emissive centers are introduced into the 2D HPs to improve the scintillation performance for the following two reasons: (1) Mn dopants in HPs snatch the energy from trap states, hence recycling the wasted nonradiative energy for scintillation;^49^ (2) The photon energy of Mn emission (~2 eV) is significantly lower than the bandgap of 2D HP host (~3.1 eV), which prevents photon loss caused by self-absorption^50^. The spectra of this series of 2D HPs are shown in Fig. 3i–l. The successful Mn incorporation is confirmed by the characteristic emission of Mn centers at ~610 nm. Another adamant evidence is the typical photoluminescence excitation (PLE) spectrum as shown in Supplementary Fig. 3, it shows that the Mn(II) emission only appears when the 2D HP host is excited. The doping procedure includes grinding and thermal treatment, which leads to ripening of the 2D HPs (Supplementary Fig. 4). The doping concentration was measured to be ~0.26–0.31% (Supplementary Table 1) by inductively coupled plasma mass spectrometry (ICP-MS), the concentration is so low that even though the ion radius of Mn^2+^ (0.97 Å) is much smaller than Pb^2+^ (1.33 Å), the integrity of 2D HP lattice structure will hardly be compromised, as is also confirmed by the XRD patterns in Supplementary Fig. 5. As a consequence, an increase of overall PL QYs by about one order of magnitude (Fig. 3m–p) to the range of 49–61% is observed. It should be noted that among 2D HPs with different halogen components, only L_2_PbBr_4_:Mn shows respectful emitting properties, while Cl-type and I-type counterparts demonstrate rather weak fluorescence. According to previous reports, this is because L_2_PbCl_4_ is intrinsically non-emissive, and the energy offset in L_2_PbI_4_ does not offer efficient exciton transfer to Mn centers^51,52^.

The scintillation performances of the Mn-doped 2D HPs are provided in Fig. 3q–t (under the irradiation of ^63^Ni source). The ^63^Ni releases β-ray with a continuous energy distribution with a maximum power of up to 66.7 keV. A gradual increase in scintillation response is found as the organic cation gets bulkier. Especially, although the PL QY (61%) of BA_2_PbBr_4_:Mn is slightly higher than that of OA_2_PbBr_4_:Mn (49%), the scintillation intensity of the former is still weaker than the latter, which highlights the effect of the organic shell in strengthening the scintillation behavior. It is noteworthy that Mn(II) scintillation is dominant but the intrinsic emissions of the 2D HPs hosts are quite weak, this result corroborates the effect of Mn(II) dopants. The emission from Mn(II) centers is also compatible with Si-based photodetectors (PDs) that sense >500 nm emissions^31^. The scintillation intensity reaches the highest in STA_2_PbBr_4_:Mn. While the PL QY of STA_2_PbBr_4_:Mn and CsPbBr_3_@Cs_4_PbBr_6_ are equal (~60%, corresponding photographs of the emitting samples are shown in Supplementary Fig. 6), the integrated scintillation intensity of the former is 17 times that of the latter, such a direct comparison powerfully confirms the advantage of the organic shell over the heavy Cs_4_PbBr_6_ shell in β-ray detection. Since β-ray can be completely absorbed within a thin surface layer, the light yield can be estimated by comparing the scintillation response with that of commercial scintillator, in this way, a high light yield of ~24,000 photons MeV^−1^ is obtained (Supplementary Fig. 7 and Suppementary Note 1)^53^.

### Aqueous synthesis and waterproofness

**Fig. 4:**
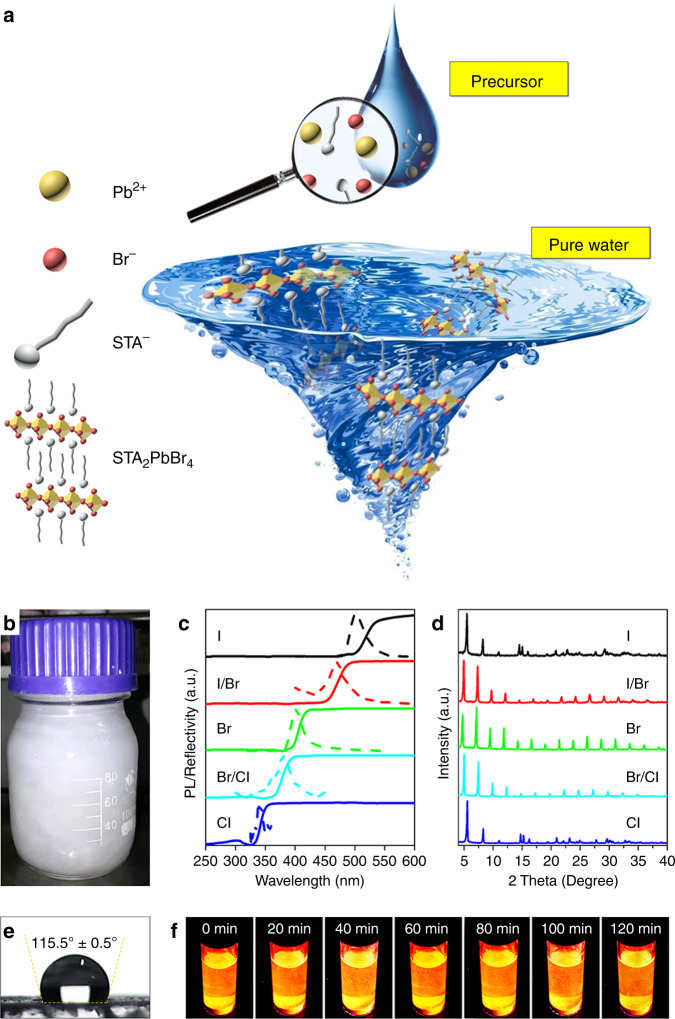
Universality of aqueous synthesis and water resistance of the 2D HP. **a** Diagrammatic presentation of the aqueous synthesis. **b** Photograph of the large-scale aqueous synthesis. **c** PL and reflectivity spectra of a series of STA-based 2D HPs with different halide component and **d** corresponding XRD patterns. **e** Contact angle test of the STA_2_PbBr_4_ film. **f** Photographs of the water treated STA_2_PbBr_4_:Mn(II) 2D HPs at different time nodes. Source data are provided as a Source Data file.

The commercial plastic scintillators suffer from stringent and labor-intensive fabrication. For example, the raw materials have to be highly pure, and the operations of cleaning and assembly before subsequent polymerization are detail-oriented, therefore greatly adding to the overall effort. The polymerization cycle lasts for several days, during which a persistent high-temperature treatment and a final cooling with precise control are needed to obtain stress-free products^54,55^. Therefore, a facile and low-cost fabrication procedure is expected when developing innovative scintillators, that’s why another unique advantage of the 2D HP scintillator, namely aqueous synthesis, seems significant. The diagrammatic presentation of the aqueous synthesis is in Fig. 4a. Referring to the well-known antisolvent-assisted recrystallization method^56^, the 2D HPs are recrystallized by pure water as antisolvent. Such facile synthesis allows for large scalability by proportional enlargement as shown in Fig. 4b, the large scale synthesis indicates ready lab-to-industry transfer. Mn-doped STA_2_PbBr_4_ by the up-scaled synthesis is offered in Supplementary Fig. 8. We found that a series of 2D HPs, including DA (*C* = 12), tetradecylamine (TA, *C* = 14), cetylamine (CA, *C* = 16), and STA (*C* = 18)-based 2D HPs, can be prepared via such aqueous synthesis, corresponding XRD patterns can be found in Supplementary Fig. 9. While organic cations with a shorter alkly chain, such as OA (*C* = 8), fail the aqueous synthesis due to a weak hydrophobicity. Moreover, the bandgap of the 2D HPs can be simply tuned by varying the halogen. Take STA-based 2D HPs as an example, the halogen-dependent PL and reflectivity spectra are presented in Fig. 4c, the results show that the overall bandgap of the STA-type 2D HPs stretch across the ultraviolet (UV) and visible (vis.) range. The XRD patterns are offered in Fig. 4d, which unveil halide-induced subtle changes in lattice. The photographs of the 2D HPs can be found in Supplementary Fig. 10. Contact angle test was then carried out to quantify the hydrophobicity induced by the organic layer (Fig. 4e), a large contact angle of 115 ± 0.5^o^ was obtained to confirm the ultrastrong water resistance (>90^o^). Consequently, not only the aqueous synthesis is enabled thereby, waterproofness is also realized to effectively protect the inner [PbBr_6_]^4−^ functional units, so that the 2D HPs are even capable of enduring long-time exposure in water. For demonstration, STA_2_PbBr_4_:Mn is directly immersed in water as shown in Fig. 4f. It was found that after harsh water treatment for 120 min, no decay in the bright 2D HPs was observed. The high water resistance is of great significance, many mature scintillators are hygroscopic, especially the well-behaved halide compounds^57,58^, but water is widely used as the coolant or reaction moderator in nuclear reactor.

### Superior performance for β-ray monitoring

**Fig. 5:**
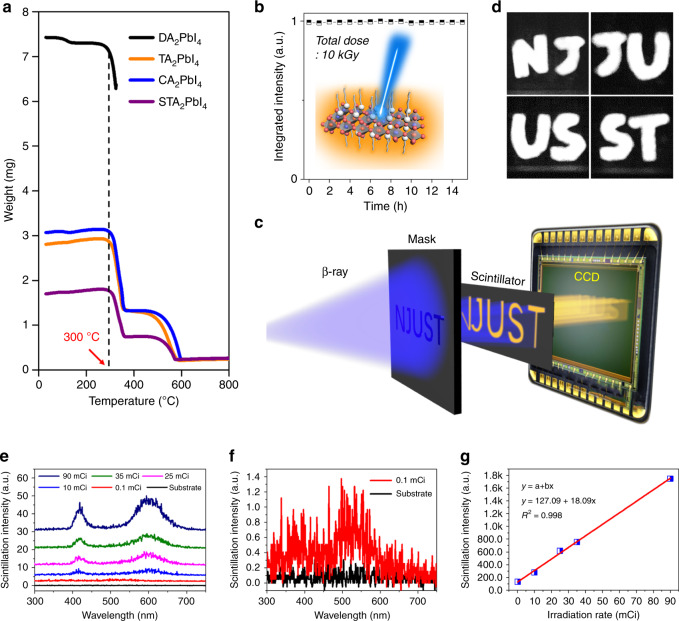
Characterizations of thermotolerance and irradiation stability. **a** TGA characterization of the 2D HPs. The decomposition temperature is ~300 °C. **b** Evolution of integrated scintillation intensity of the Mn-doped 2D HPs during long-time irradiation test. Inset: schematic view of the scintillation behavior of the Mn-doped 2D HPs. **c** Illustration of the setup of the system for scintillation test. **d** The obtained patterns. **e** The scintillation spectra of the 2D HP scintillator under different β-ray irradiation intensity. **f** The scintillation spectrum under the irradiation activity of 0.1 mCi. **g** Linear response of the 2D HP scintillator down to the detection limit. Source data are provided as a Source Data file.

When used for radiation monitoring in nuclear plants, good thermotolerance is crucial for β-ray scintillators, because β-ray detection technologies are short-range detection technologies owing to the moderate penetration power of β-ray, and good thermostability ensures a long service lifetime. Typically, the temperature of the cold inlet end in a nuclear reactor reaches >230 °C, the temperature of the nearby air may be lower, but still sets a great challenge for β-ray scintillators. Rare commercial organic scintillators can withstand such high temperatures. Take the mainstream plastic scintillators as an example, the common bases of PS, PMMA or PVT undergo a glass transition at <105 °C, above which the plastic scintillators completely fail. To test the thermotolerance of the 2D HP scintillators, thermogravimetry (TGA) characterizations were carried out. As shown in Fig. 5a, not until 300 °C the decomposition takes place (Table 2), which is caused by the evaporation of the organic components^59,60^. The further weight loss caused by PbI_2_ evaporation occurs at ~600 °C. The in situ fluorescence at elevated temperatures was also measured (Supplementary Fig. 11), the result shows that even when the temperature reaches beyond 110 °C, at which point commercial plastic scintillators already fail owing to a glass transition of the bases (<105 °C), STA_2_PbBr_4_:Mn still retains 34% of the overall integrated PL intensity, which corresponds to a light yield of approximately 8100 photons MeV^−1^.

**Table 2:** Comparison between the decomposition temperatures of typical commercial plastic scintillators and that of the 2D HP scintillators.

Scintillator	Decomposition point (°C)
Plastic scintillator _(base materials: PS, PMMA, and PVT)_	70–105
2D HPs (in this work)	300

Radiation damage is another inevitable issue that all scintillation materials suffer from. In general, the intense irradiation would induce damage to the crystal lattice, which brings a reduction in the output of scintillators caused by the creation of damage centers. The damage centers not only dramatically increase non-radiative recombination, but also absorb the scintillation light^61,62^. For commercial plastic scintillators, high-energy rays can do harm to both the fluorescent molecules and the bases, therefore compromising the scintillation performance^55^. 2D HPs are renowned for dramatically enhanced chemical stability compared with the parental 3D counterparts^63^, this is because the involvement of organic cations strengthens the lattice owing to appreciable van der Waals interactions^43,64,65^. To further evaluate the irradiation stability of the 2D HPs as β-ray scintillators, a test was conducted on STA_2_PbBr_4_:Mn as an example. Figure 5b displays the long-term stability under continuous β-ray irradiation with an accumulated dose reaching up to 10 kGy (dose rate 0.67 kGy h^−1^). This radiation dose is >10^6^ times larger than that in brain computed tomography (CT, typically ~8.6 mSv per time by X-ray). The result unambiguously confirms that the STA_2_PbBr_4_:Mn is able to offer a persistent scintillation performance, corroborating a robust scintillation behavior. Besides, no radiation drift, i.e., an enhancement or decrease of scintillation light yield after a large amount of radiation exposure, was observed after the irradiation test, corresponding scintillation spectra can be found in Supplementary Fig. 12.

Proof-of-concept radiation test is then demonstrated to show that the 2D HP scintillator offers effective and highly discernable response to β-ray. The set-up of a self-built measurement system is shown in Fig. 5c. The incident β-ray is firstly shaped by masks of a series of hollowed-out letters “NJ”, “JU”, “US”, and “ST” (Supplementary Fig. 13) before working on the 2D HP active layer, the produced scintillation is then captured by an EMCCD camera. Clearly, the obtained patterns perfectly reproduce these letters in masks (Fig. 5d), while there is no fluorescence signal in the nonirradiated region. Further, afterglow is evaluated with time-resolved decay of emitting photons^66^. As shown in Supplementary Fig. 14, the decay time is 0.5 μs, and the relative afterglow intensities at 5 μs, 6 μs, and 7 μs are 5.77‰, 2.18‰, and 1.26‰, while for comparison the characteristic afterglows of CsI:Tl and LYSO for X-ray reach several milliseconds and several hours, respectively^67,68^. Detection limit is another core parameter of scintillator detector, the measuring setup is illustrated in Supplementary Fig. 15 and Supplementary Note 2, the irradiation activity of the β-ray source was gradually reduced until a distinguishable scintillation response was not observed, the spectra are shown in Fig. 5e, f. The integration intensity of the scintillation response was linearly proportional to the irradiation activity, and the limit was determined to be 0.1 mCi (3.7 × 10^6^ Bq, Fig. 5g). To get an idea of how small the detection limit is, we refer to the file of “WS 533-2017 protection requirements on nuclear medicine patient”, it states that the irradiation activity must be <2 × 10^8^ Bq (pure β-ray by ^89^Sr in medicine) before the patient can be discharged, it means even in such an unoptimized measuring setup, the low-detection limit 2D HP scintillator already meets the requirement of medical diagnosis. The irradiation activity of nuclear irradiation is much more intensive compared with radioactive medicine, the great potential of 2D HP scintillator for nuclear monitoring can therefore be confirmed.

## Discussion

A type of β-ray scintillator based on 2D HPs is demonstrated. The design principle of a high-performance β-ray scintillator is discussed, according to which comprehensive composition engineering is carried out. The optimization of organic cations empowers efficient scintillation by greatly enhancing the capturing efficiency of β particles, and the extrinsic Mn(II) dopants improve the scintillation performance via serving as emitting centers with no self-absorption. As a result, effective β-ray scintillation performance is obtained, and no functionality decay or hysteresis is observed after being exposed to an accumulated radiation dose of 10 kGy (dose rate 0.67 kGy h^−1^). Moreover, the 2D HPs also enjoy low-cost aqueous synthesis along with superior waterproofness, which is ready for a lab-to-fab transfer. It is believed that this work will open new possibilities of HPs towards high-performance β-ray detection for nuclear radiation monitoring.

## Methods

### Chemicals

PbI_2_ (Aladdin, 98%), PbBr_2_ (macklin, 99%), PbCl_2_ (Aladdin, 99.99%), stearamine (STA, 90%, J&K Scientific Ltd.), cetylamine (CA, Aladdin, 98%), tetradecylamine (TA, ≥ 95%, TGI Lot.), dodecylamine (DA, Aladdin, 98.0%), octylamine (OA, Aladdin, 99%), butylamine (BA, Aladdin, 98%), hydroiodic acid (meryer, 47%), hydrobromic acid (meryer, 48%), hydrochloric acid (36–38%, Shanghai lingfeng chemical reagent co. LTD), dodecylamine iodide (DAI, Xi’an p-OLED Corp.), acetic acid (≥99.5%, Nanjing chemical reagent co. LTD), and ethyl alcohol (99.7%, SCRC), MnBr_2_ (98%, Aladdin)

### Fabrication of (STA)X/(CA)X/(TA)X/(DA)X/(OA)X/(BA)X

We will take (STA)I for example, and the others follow the same procedures. STA was dissolved in 300 ml acetic acid, then HI/water solution was added under vigorous stirring. STA should be excessive in this reaction. The solution immediately turned white turbid owing to the formation of (STA)I (It is noteworthy that because HI is highly instable when exposed to light, the reaction was taken in deep-brown vessel for protection, but HBr and HCl are much more stable to light). The (STA)I was immediately extracted by 10,000 × 3 min centrifugation and washed using pure acetic acid, the centrifugation-washing cycle was repeated several times until the supernatant became transparent and the (STA)I precipitates became totally white. It should be noted that for DAX, OAX and BAX, diethyl ether rather than acetic acid was used for washing. After that, the (STA)I was dissolved in ethyl alcohol and reprecipitated by rotary evaporation under lowered pressure.

### Aqueous synthesis of the 2D HPs

We will take STA_2_PbI_4_ for example, and the others basically follow the same procedures. 2:1 (STA)Br and PbBr_2_ were dissolved in DMSO to form precursor with a high concentration of 0.5 M. In all, 0.5 ml of the precursor was injected into 20 ml deionized water under vigorous stirring. The solution immediately turned white turbid owing to the formation of STA_2_PbBr_4_. After that, a centrifugation at 5000 rpm × 2 min was carried out, the precipitates were dried under a low pressure for further characterizations. For Mn-doping, the norminal doping ratio of Mn is 10%, the methods follows the previous report by Biswas et al.^50^. The 2D HPs were directly grounded with MnBr_2_, and then annealed at 100 °C in a nitrogen atmosphere for 30 min.

### Preparation of BA_2_PbBr_4_ and OA_2_PbBr_4_

The BA_2_PbBr_4_ and OA_2_PbBr_4_ cannot be prepared by the above aqueous synthesis, because they are not water-resistant. Taking BA_2_PbBr_4_ for example, 2:1 (BA)Br and PbBr_2_ were dissolved in DMSO to form precursor with a high concentration of 0.5 M. In total, 0.5 ml of the precursor was injected into 20 ml toluene under vigorous stirring. Then 1 ml water was added to trigger the formation of the BA_2_PbBr_4_^60^. After that, a centrifugation at 5000 rpm × 2 min was carried out, the precipitates were dried under a low pressure for further characterizations.

### Characterizations

XRD measurements were carried out with a Bruker D8 Advance XRD system. SEM images were taken by FEI field emission electron microscope, Quanta 250 F. PL spectra are measured by Cary Eclipse Fluorescence Spectrophotometer. The reflectance spectra are measured by SHIMADZU UV-3600 UV-VIS-NIR spectrophotometer. The time-resolved PL spectra were measured using self-built setup with single-photon counter, the excitation wavelength is 375 nm. The contact angle was measured using a contact angle meter. Inductively coupled plasma mass spectrometry (ICP-MS) data were obtained by employing Agilent 7700. Thermogravimetric analysis (TGA) measurements were recorded using Perkin Elmer STA 6000. The temperature-dependent fluorescence spectra were measured using a PG 2000 fiber optic spectrometer. The afterglow curves were measured by using a pulse 375 nm excitation (pulse duration of 50 ps), and were recorded in oscilloscope mode using a SPCM-AQRH-15 APD detector, the 2D perovskite scintillator was placed in total darkness for 24 h before the afterglow measurement was carried out.

### β-ray scintillation test

2D HPs in the form of powers were carefully grinded to be very fine, which was then covered onto wide tapes until complete adherence. The tapes were then cut into masks for subsequent β-ray scintillation test. The scintillation spectra were obtained using a Cary Eclipse Fluorescence Spectrophotometer in a PMT detection mode.

### Estimation of the backscattered coefficient *η* of the organic shell of 2D DA_2_PbBr_4_

The Eq. 3 *η* = *lnZ/6−1/4 (Z* ≥ *10)* in the main text does not apply to elements with *Z* < 10, including C (*Z* = 6), H (*Z* = 1), and N (*Z* = 7) in the organic shell. A previous report revealed that with an electron beam energy of 5 keV, the *η* of C is <5%, the *η* values of H and *Z* is even lower. And as the energy of incident particles increases, the *η* of C, H, N further decreases, while the *η* of high-*Z* elements increases. Taking the *η* of C, H, N to be 5%, the $\bar \eta$ of the organic shell is evaluated as 5%, which is larger than the practical situation^69^.

### Discussion on the elastic and inelastic scattering effect

The elastic scattering cross-section (*σ*_*N*_*(θ)*) can be expressed as:

4 $$\sigma _N\left( \theta \right) = \pi \, * \,\left( {Z\, * \,e/\left( {V\, * \,\theta } \right)} \right)^2$$

where *Z* is the nuclear charge number, *e* is elementary charge, *V* is the kinetic energy of β particles, and *θ* is the scattering angle. Similarly, the scattering cross section (*σ*_*e*_*(θ)*) of inelastic scattering can be quantified as:

5 $$\sigma _e\left( \theta \right) = Z\pi \, * \,\left( {e/\left( {V\, * \,\theta } \right)} \right)^2$$

The cross section represents the probability that a scattering event will take place. Thus, the probability (*Z*/(1 + *Z*)) of elastic scattering is *Z* times larger than that of inelastic scattering (1/(1 + *Z*)).

## Supplementary information

Supplementary Information

Peer Review File

## Data Availability

The data that support the findings of this study are available from the corresponding author upon reasonable request. The source data underlying Figs. 3e–l, q–t, 4c–d, and 5a–g, and Supplementary Figs. 1, 2, 3, 5a–d, 7, 9, 11, and 14 and Supplementary Table 1 are provided as a Source Data file. Source data are provided with this paper.

## Source data

Source Data
