# Supplementary material for: Two-dimensional halide perovskite as β-ray scintillator for nuclear radiation monitoring
Source: Nat Commun. 2020 Jul 7;11:3395. doi: 10.1038/s41467-020-17114-7 (PMC7341884; doi:10.1038/s41467-020-17114-7)
Supplement: Supplementary file 1 — Supplementary Information [file 41467_2020_17114_MOESM1_ESM.pdf]

Supplementary Information

Two-dimensional halide perovskite as β-ray scintillator for nuclear radiation monitoring

Yu *et al.*

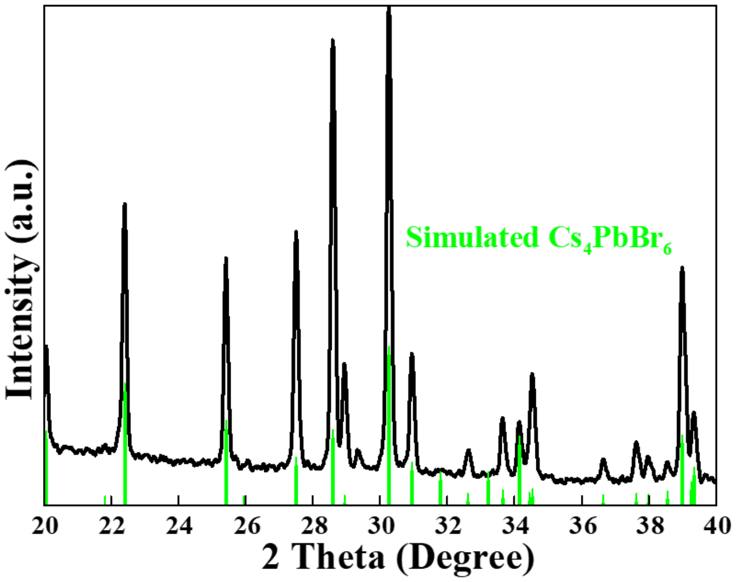

Supplementary Figure 1. The XRD pattern of $CsPbBr_3$@Cs_4PbBr_6 powders. Source data are provided as a Source Data file.

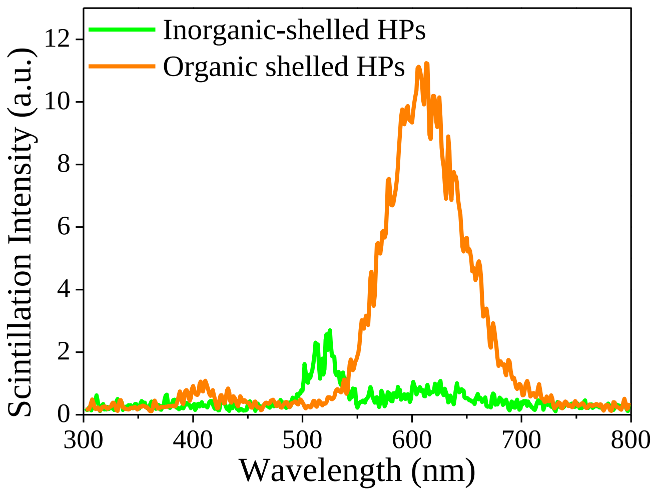

Supplementary Figure 2. Scintillation response of inorganic-shelled and organic shelled HPs towards β ray. The 2D system refers to DA_2PbBr_4:Mn (DA is dodecylamine). Source data are provided as a Source Data file.

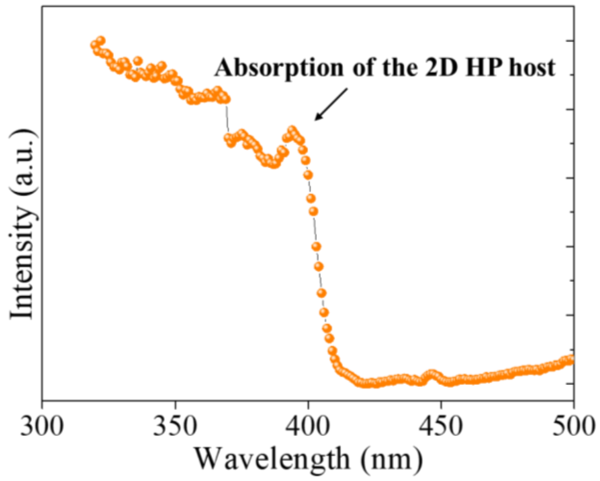

Supplementary Figure 3. Typical PLE spectrum of the Mn-doped 2D HPs (STA_2PbBr_4). The tracing emission is at 610 nm, *i.e.*, the emission of Mn(II) centers. Source data are provided as a Source Data file.

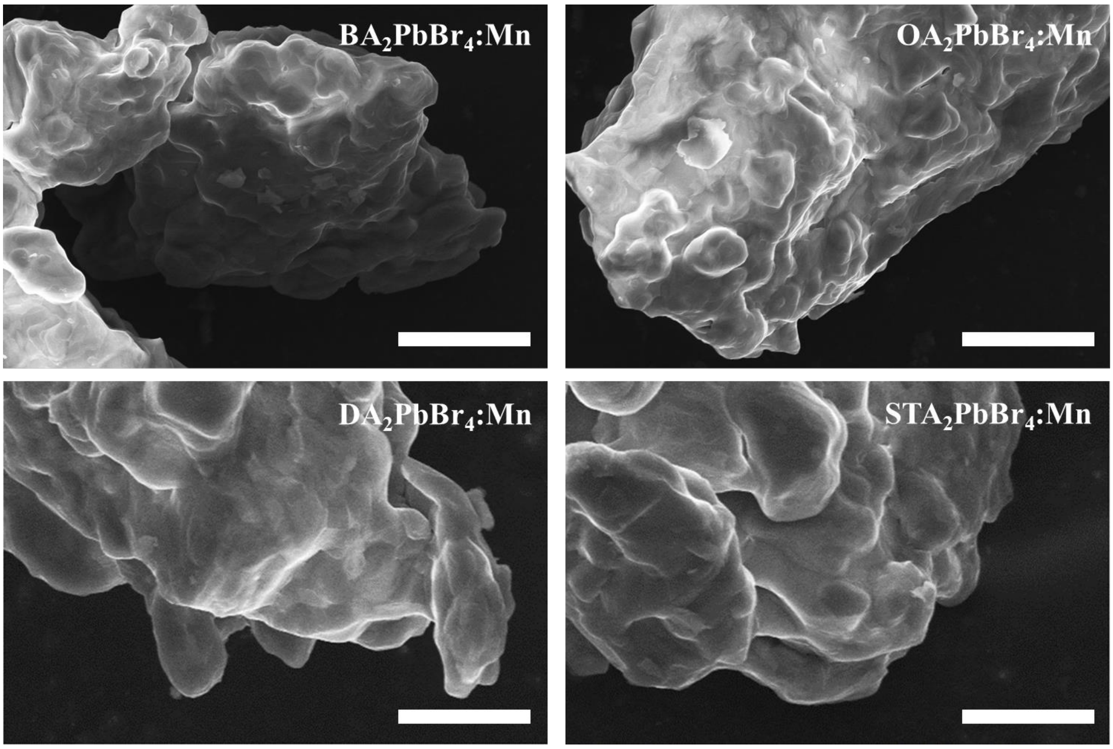

Supplementary Figure 4. SEM images of BA_2PbBr_4:Mn, OA_2PbBr_4:Mn, DA_2PbBr_4:Mn and STA_2PbBr_4:Mn, respectively, after the Mn doping procedures, including grinding and annealing (100 degrees Celsius for 20 mins in nitrogen). The scale bars are 10 μm.

Supplementary Table 1. The nominal doping concentration of Mn.

Material	Doping concentration
BA_2PbBr_4:Mn	0.29%
OA_2PbBr_4:Mn	0.26%
DA2PbBr4:Mn	0.31%
STA_2PbBr_4:Mn	0.27%

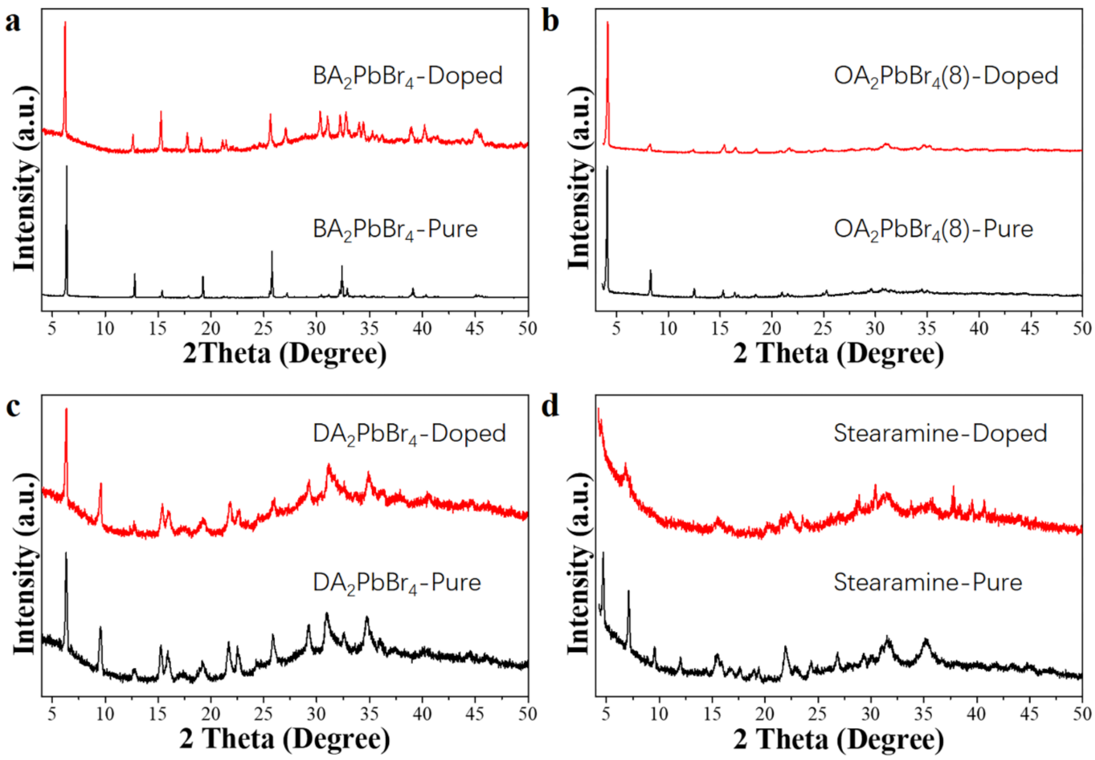

Supplementary Figure 5. XRD patterns of a series of pristine and Mn(II)-doped 2D HPs. Corresponding organic components are (a) butylamine (BA), (b) octylamine (OA), (c) dodecylamine (DA), (d) stearamine, respectively. Source data are provided as a Source Data file.

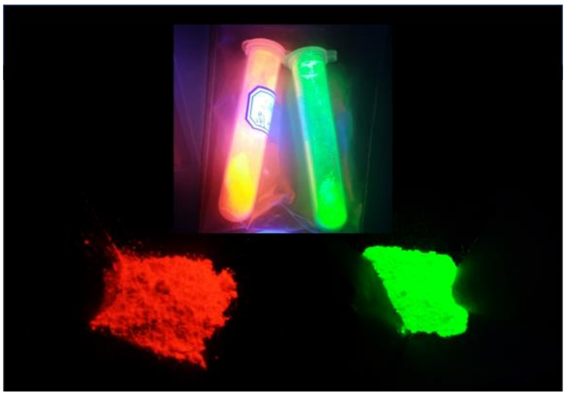

Supplementary Figure 6. Photographs of the emitting STA_2PbBr_4:Mn and $CsPbBr_3$@Cs_4PbBr_6.

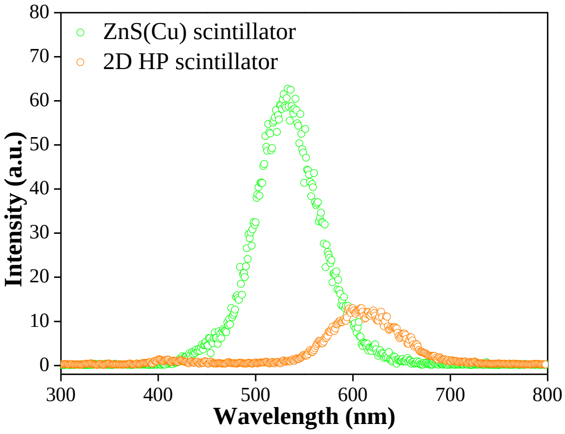

Supplementary Figure 7. Scintillation spectra of ZnS(Cu) and STA_2PbBr_4:Mn. Source data are provided as a Source Data file.

Supplementary Note 1: Since β ray can be completely absorbed within a thin surface layer, the light yield can be estimated by comparing the scintillation response with that of commercial scintillator.[1] Here ZnS:Cu (with a high light yield towards β ray of ~100000 photons MeV^{-1}) as one of the most efficient scintillator towards β ray sensing is adopted as the reference.[2-4] One of the most important reason for choosing ZnS(Cu) is that it is in the form of powder the same as the 2D perovskite scintillator here, all the processing can be kept the same for an accurate reference. Corresponding scintillation response is shown as **Supplementary Figure 7** in the following. The integrated scintillation intensity of the STA_2PbBr_4:Mn scintillator is 24.7% of that of ZnS(Cu), therefore, the light yield of the 2D perovskite scintillator is estimated to be >24000 photons MeV^{-1}. This value is even higher than some typical organic scintillators including naphthalene (~15000 photons MeV^{-1}) and diphenyl (~23000 photons MeV^{-1}).

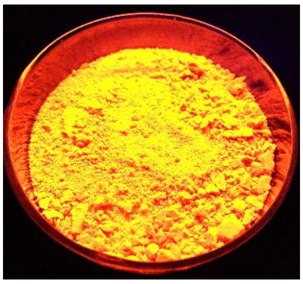

Supplementary Figure 8. Large-scale synthesis of Mn-doped STA_2PbBr_4.

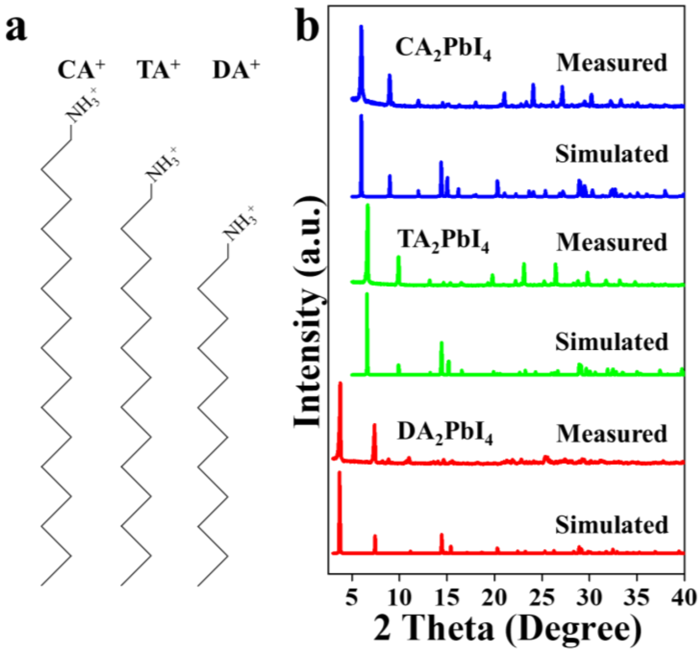

Supplementary Figure 9. (a) Diagrammatic presentation of the structure of dodecylamine (DA), tetradecylamine (TA) and cetylamine (CA). (b) XRD patterns of DA_2PbI_4, TA_2PbI_4 and CA_2PbI_4 by the aqueous synthesis. Source data are provided as a Source Data file.

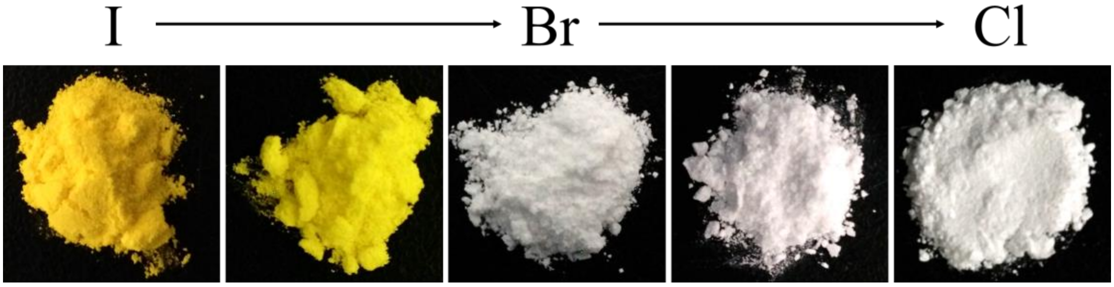

Supplementary Figure 10. Photographs of STA-based 2D HPs with different halide by the aqueous synthesis.

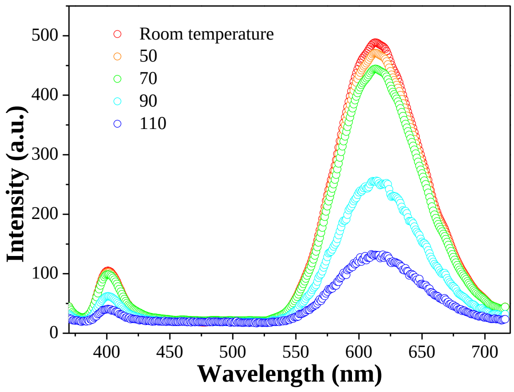

Supplementary Figure 11. Temperature-dependent PL spectra of Mn:STA_2PbBr_4. Source data are provided as a Source Data file.

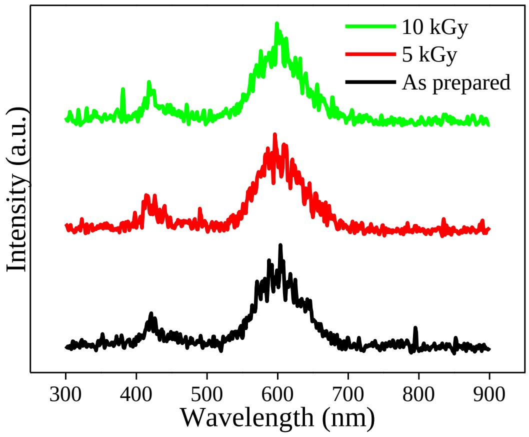

Supplementary Figure 12. The evolution of scintillation spectra of STA_2PbBr_4: Mn during the irradiation test. Source data are provided as a Source Data file.

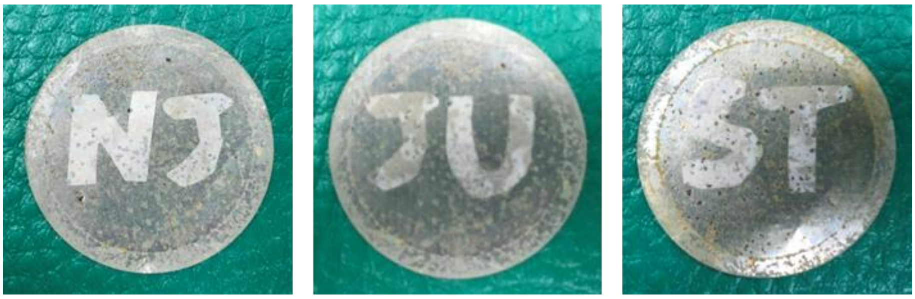

Supplementary Figure 13. The masks for β ray imaging.

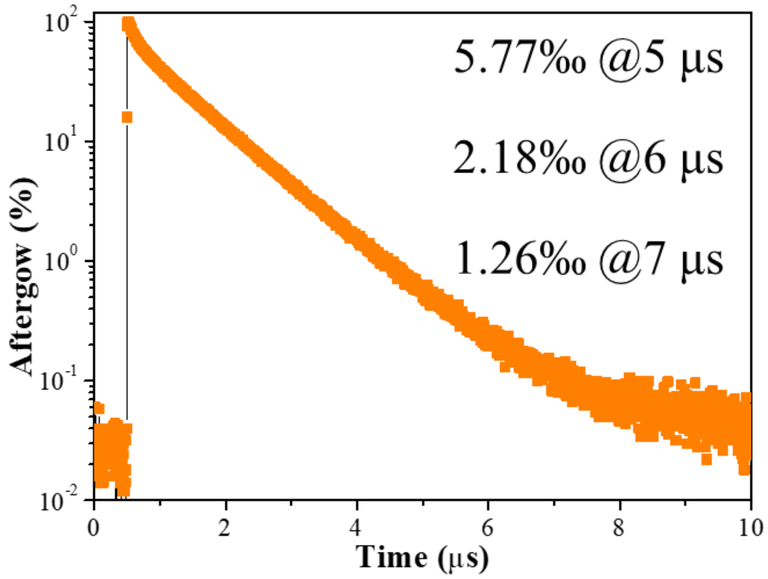

Supplementary Figure 14. Afterglow of STA_2PbBr_4:Mn after cutoff of excitation. Source data are provided as a Source Data file.

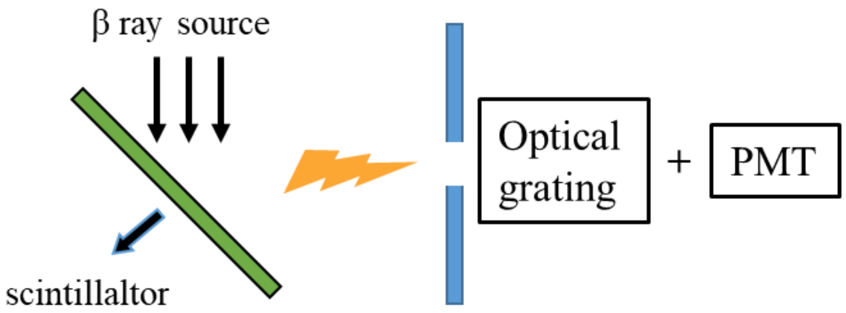

Supplementary Figure 15. The measuring setup for the detection limit.

Supplementary Note 2: It should be noted that the detection limit of a scintillator detector is not an absolute value, it is highly related to the matching degree of fluorescence wavelength and photomultiplier tube, and is also determined by the exposure time, the longer the exposure time, the lower detection limit. Therefore, the detection limit is only meaningful when compared with reference scintillators. Since the emission peak of most commercial scintillator plastic for β ray detection is within the ultraviolet range, which is quite different from the 2D perovskite scintillator (~610 nm), ZnS:Cu with an emission peak of 530 nm is used as the reference scintillator. One thing we should know about ZnS:Cu is that is offers a quite high physical light yield (~100000 photons MeV^{-1}), i.e., the fraction of the absorbed radiation energy transformed into light, but the practical light yield, defined as the amount of light obtainable from a given intensity of radiation, is greatly compromised by the strong self-absorption, because the scintillation is produced in the front of scintillator and has to pass through it to reach the PMT in the rear,[2] the measuring setup was designed as shown in Supplementary Figure 15, the ZnS:Cu was excited by β ray to produce scintillation that was directly measured without passing through the ZnS:Cu scintillator, the detection limit of 2D perovskite scintillator was measured the same way for comparison. In this way, the detection limit is measured to be 0.1 mCi, namely 3.7×10^{6} Bq, comparable to that of ZnS:Cu (1.5×10^{6} Bq).

Supplementary References

1. Mykhaylyk, Vitaliy B., Kraus, Hans, Saliba, Michael. Bright and Fast Scintillation of Organolead Perovskite $MAPbBr_3$ at Low Temperatures. *Mater. Horiz.* **6,** 1740-1747 (2019).
2. Kallmann, Hartmut. Quantitative Measurements with Scintillation Counters. *Phys. Rev.* **75,** 623-626 (1949).
3. Xu, Zhi-Heng, Tang, Xiao-Bin, Hong, Liang, Liu, Yun-Peng, Chen, Da. Structural effects of ZnS:Cu phosphor layers on beta radioluminescence nuclear battery. *J. Radioanal. Nucl. Ch.* **303,** 2313-2320 (2014).
4. Russo, J., Litz, M., Ray, W., 2nd, Smith, B., Moyers, R. A radioluminescent nuclear battery using volumetric configuration: (63)Ni solution/ZnS:Cu,Al/InGaP. *Appl. Radiat. Isot.* **130,** 66-74 (2017).
