# Supplementary material for: Two-dimensional halide perovskite as β-ray scintillator for nuclear radiation monitoring
Source: Nat Commun. 2020 Jul 7;11:3395. doi: 10.1038/s41467-020-17114-7 (PMC7341884; doi:10.1038/s41467-020-17114-7)
Supplement: Supplementary file 2 — Peer Review File [file 41467_2020_17114_MOESM2_ESM.docx]

Peer Review Information

**Journal:** Nature Communications

**Manuscript title:** Two-dimensional halide perovskite as β-ray scintillator for nuclear radiation monitoring

**Corresponding author name(s):** Prof Haibo Zeng

**Editorial notes:**

**none**

**Reviewer comments & decisions:**

**Reviewer comments, first version:**

Reviewers' comments:

Reviewer #1 (Remarks to the Author):

Zeng et al reported the “two-dimensional (2D) halide perovskite materials as β-ray scintillator for nuclear radiation monitoring”. Technically, the authors only show a β-ray induced luminescence phenomenon, which is far away from the detection or monitor. They claimed the 2D halide perovskites demonstrated the superior irradiation hardness, thermotolerance and superior waterproofness, which are suit for the high performance β-ray detection. However, the contents were badly organized and some key points are less evidential and misleading. Besides, the issues of greater concern in the β-ray detection community have not been clarified. To be honest, this paper is more suitable for a professional journal, related with light or luminescence.

1. The paper used several pages (from page 6, line 118 to page 9, line 198) to explain why should use light elements benefit for stopping β-ray. However, many classical theories can be able to find in the book of “Radiation detection and measurements, G.K. Knoll, 2000”. Here the corresponding content is not necessary. Besides, there is no specific and clear conclusion. Although there are light elements such as C, H, there are also heavy elements such as Pb, I in the hybrid materials. Which dominate the capture cross section of 2D HP? The calculated capture cross section should be compared with that of the typical scintillator materials.
2. The calculated backscattered coefficient for Cs4PbBr6 is 45.4% and that of DA2PbBr4 (C24H54N2PbBr4) is 28.9%. The QY of DA2PbBr4 is 55% and that of CsPbBr3@Cs4PbBr6 is 60%. However, the result shows the scintillation response of the former is ＞10 time larger than that of the latter. From the results, it demonstrates the light elements are not the key point reason as β-ray scintillator detectors. What’s the difference of organic-shelled and inorganic-shelled?
3. Line 231, a gradual increase in scintillation response is found as the organic cation gets bulkier. (BA is better than OA.) Therefore, what is the mainly effects between the Cs and organic? For low Z-elements and bulk volumes, which is on the dominated?
4. The environmental stability of Cl-based 2D HPs is usually better and with low-Z element compared with I or Br. What’s the advantage of I-based and Br-based materials as β-ray scintillator?
5. As a β-ray scintillator detector, either the detection limitation or the pulse height spectra of isotype (like 63Ni@66.7keV) with the full energy peak should be given. These are two core parameters for evaluating the hard nuclear radiation detector.
6. Compared with the typical scintillators such as CsI, LYSO, what is the light yield of 2D HPs scintillator?
7. The authors claim “commercial plastic scintillators is the relatively high cost”, this is also contrary to the facts. For the counter type β-ray scintillator detector, the price is mainly determined by Si-diode or PMT and readout circuit.
8. The authors claim the quite low QYs for pure 2D HPs (＜8%), but the Mn(II) doped BA2PbBr4, OA2PbBr4, DA2PbBr4, STA2PbBr4 were all significantly enhanced. However, the radius of Mn is less than Pb and there is a large difference in atom size, which affects the crystal structure. What about other divalent cation? What’s the doping concentration in these samples?
9. The peak positions for both undoped sample (shown in Fig. S2) and doped samples are all in the same wavelength ~610 nm. As the authors mentioned the trap states of Mn(II) were response for the enhanced luminescence, the position should change before and after doping. In fact, the luminescences come from the self-trap states from the PbI6 octahedron is usually found in low dimensional halide perovskites. Therefore, the corresponding discussion is misleading. Besides, the intensity of luminescence is also influenced by the crystalline quality of samples, from the pictures in Fig. 3(a)-(d), however, which is ignored by the authors.
10. How the hydrophobicity of organic cations in 2D HPs determine whether it can be synthesized by water?
11. There is only one peak in Fig. 4(c) the PL spectra, which is not consistent with the pure 2D halide perovskites shown in the manuscripts (Fig. S2).
12. In terms of thermotolerance, however, the stability of phase or composition can’t totally represent the working stability in high temperature. The performance of the scintillators in high temperature under the illumination of β-ray is critically required, for example, light yield, decay time, and so on. The data in Fig. 5(b) looks beautiful, but is quite limited and the accuracy of ordinate is very poor.
13. If I understand the result in Fig. 5(c) correctly, the imaging shows the area with 2D HPs scintillator, which do not help for the real application. What’s the space resolution?
14. The afterglow effect is very important for imaging using scintillator detector. The 2D HPs result should compare with the typical scintillators, like LYSO, CsI. Besides, the time response (resolution) should be added in the manuscript.
15. In Fig. S9, the authors show the comparisons of the PL spectra before and after the radiation tests, however, the curves have very week SN ratio, which is insufficient for claim the sample with superior irradiation hardness.


Reviewer #2 (Remarks to the Author):

This paper presents a breakthrough in my mind. Perovskites for beta-ray scintillation is a totally new idea. The materials tested are very well selected and designed for optimized scintillation. The stability of the perovskites in water is impressive. The only important parameter I miss in this paper is the detection limit, which should be provided in Bq/min and added for the various materials tested.

**Author rebuttal, first version:**

**Response to Reviewer #1**

Zeng *et al* reported the “two-dimensional (2D) halide perovskite materials as β-ray scintillator for nuclear radiation monitoring”. Technically, the authors only show a β-ray induced luminescence phenomenon, which is far away from the detection or monitor. They claimed the 2D halide perovskites demonstrated the superior irradiation hardness, thermotolerance and superior waterproofness, which are suit for the high performance β-ray detection. However, the contents were badly organized and some key points are less evidential and misleading. Besides, the issues of greater concern in the β-ray detection community have not been clarified. To be honest, this paper is more suitable for a professional journal, related with light or luminescence.

**Response:** Dear Reviewer, thank you so much for your time and the valuable comments, and we have carefully revised the manuscript accordingly to enhance the manuscript, we hope the new version of manuscript addresses your concerns. All the revisions in the manuscript and in this reply file have been highlighted in yellow.

It is commented that “**…Technically, the authors only show a β-ray induced luminescence phenomenon, which is far away from the detection or monitor…However, the contents were badly organized and some key points are less evidential and misleading…**”. In our opinion, scintillators refer to a class of materials that emit fluorescent signals when excited by high-energy rays, it is exactly β-ray induced luminescence phenomenon in principle, and we have enhanced the performance characterizations of the scintillator according to the comments. We also assume that you may have major misunderstanding of some of the contents as we carefully go through your comments, and we have explained in detail in the following response as well as to reorganize the content, we sincerely hope the response addresses your concerns.

It is also commented that “**…Besides, the issues of greater concern in the β-ray detection community have not been clarified…**”. There probably are some concerns in the β-ray detection community that have not been reached in this work, however, the aim of this work is to introduce a kind of 2D perovskite scintillator with high light yield, low detection limit, irradiation robustness, water-resistance and thermostability, coupled with the facile chemical route towards it. These properties allow the 2D perovskite scintillator to cost-effectively monitor β ray irradiation intensity in some harsh environment that easily fail commercial β ray scintillators, such as nuclear plant.

At last, we believe the results in this work may cheer up the community of halide perovskite. Halide perovskites are a type of cheap semiconducting material with superior optoelectronic and optical properties, but also suffer from poor stability when considered for real-life applications (solar cells, light emitting diodes, *etc.*). Worldwide researchers in this filed have been asking what we can practically do with this material. Very recently, several groups have proposed the application of halide perovskite as sensitive materials (either in a direct way as photoconductive detectors or in an indirect way as scintillators) for high-energy rays, including Gamma ray and X-ray.^1-8^ Later, it was found that halide perovskites are extremely stable against these high-energy rays.^9-15^ All these results make high-energy rays detection one of the most promising ends for halide perovskites, and this work for the first time introduces their functionality in detecting β ray as a missing supplement, which we believe is therefore of great positive significance.

Detailed response is listed as follows, please check.

1. The paper used several pages (from page 6, line 118 to page 9, line 198) to explain why should use light elements benefit for stopping β-ray. However, many classical theories can be able to find in the book of “Radiation detection and measurements, G.K. Knoll, 2000”. Here the corresponding content is not necessary. Besides, there is no specific and clear conclusion. Although there are light elements such as C, H, there are also heavy elements such as Pb, I in the hybrid materials. Which dominate the capture cross section of 2D HP? The calculated capture cross section should be compared with that of the typical scintillator materials.

**Response:** As suggested, we have according to your comment condensed and reorganized this part, the **Discussion on the elastic and inelastic scattering effect** has been moved to the supporting information (as can be checked in the revised supporting information file). It is commented that the theory introduction part is unnecessary because it is already mature. We agree that there is indeed existing classic theory for reference, and we appreciate a lot for the recommendation of the book. However, we do not mean to simply copy the theory to fill in the manuscript, we also hope you can understand that the huge community of halide perovskite has got used to the concise X-ray/gamma ray absorption theory that emphasizes the effect of heavy elements, and proper foreshadowing is necessary when introducing a different, or even somewhat contrary, rationale of β ray scintillator with stress on light elements. Therefore, we have added corresponding statement (page 6 in the revised manuscript):

“…It will be useful to present a simplified view of how the β ray interacts with scintillators…”

It is commented that there is no conclusion on which element dominates the capture cross section of 2D halide perovskites. In our opinion, for individual elements in compounds, there is only the concept of a scattering cross section, not a capture cross section, because individual elements do not scatter β particles does not mean they absorb β particles. For instance, the light organic component (comprised on C, H, and N) owns the smallest η (backscattered coefficient) in 2D perovskites (<5%, which means elastic scattering is very weak between the organic component and β particles, the inelastic is Z times smaller than elastic scattering, therefore, the overall interaction between β ray and the organic component is quite weak. Consequently, β particles would go through the organic layer to the [PbBr_6_] functioning units to produce scintillation response rather than to be absorbed by the C, H, N elements.

It is also commented that the calculated capture cross section should be compared with that of the typical scintillator materials. In fact, the BSE coefficient *η* of various kinds of typical scintillators has already been listed in the previous manuscript (Figure 2e in the manuscript) as shown in the follows:

**Figure R1.** Inset: comparison of the backscattered efficiency of several typical scintillators (Figure 2e in the previous manuscript).

2. The calculated backscattered coefficient for Cs_4_PbBr_6_ is 45.4% and that of DA_2_PbBr_4_ (C_24_H_54_N_2_PbBr_4_) is 28.9%. The QY of DA_2_PbBr_4_ is 55% and that of CsPbBr_3_@Cs_4_PbBr_6_ is 60%. However, the result shows the scintillation response of the former is >10 time larger than that of the latter. From the results, it demonstrates the light elements are not the key point reason as β-ray scintillator detectors. What’s the difference of organic-shelled and inorganic-shelled?

**Response:** It is supposed that you mean the less than 2-fold decrease of the backscattered coefficient does not match with the >10 fold increase of scintillation response. In fact, in addition to the improving of β ray capturing efficiency, incorporating light organics to form 2D perovskites also increase the scintillation response by suppressing the competing bremsstrahlung effect. When inelastic scattering occurs, the energy of β particles is transferred to the scintillator, producing a series of electron signals and light signals, the light signals include X-ray and visible phonons (the scintillation response). Bremsstrahlung effect is the cause of continuous X ray signal, *i.e.*, X rays are released when high-speed charges suddenly accelerate or decelerate. The intensity of bremsstrahlung is proportional to Z^2^, which means a weakened bremsstrahlung effect with light elements to in contrary enhance the scintillation effect. Since X-ray is high-energy, fluorescence is low-energy, the compensation to fluorescence response would be considerable. Accordingly, we have added this content in the revised manuscript (page 8 to page 9 in the revised manuscript):

“…After β particles are absorbed by 2D HPs, the incorporation of light elements also enhances the scintillation response by suppressing energy allocation on useless signals. Specifically, the photonic signals by β ray excitation include high-energy X-ray and low-energy fluorescence (the scintillation response). Bremsstrahlung effect is the cause of continuous X ray signal, *i.e.*, the energy of β particle is consumed by releasing continuous X rays when high-speed charges suddenly accelerate or decelerate. The intensity of bremsstrahlung is proportional to Z^2^, which means a weakened bremsstrahlung effect with light elements. The $\bar{Z}$ of compounds is given by eq 3:

$\bar{Z}$=∑(NZA)/ ∑(NA)	eq 3

where N is the number of atoms of each element with atomic weight A and atonic number Z. The $\bar{Z}$ values of 0D and 2D HP systems are calculated to be 43 and 33.6, which indicates that 39% energy consumption of high-energy X ray will be reduced to compensate the competing low-energy fluorescence signal…”

It should be noted that we do not think it is a proper way to analyze the outcome in a completely quantitative way, because the interaction between β ray and scintillator is very complex, a comprehensive prediction in physics can easily deviates from the experiment results. We suppose qualitative analysis is a more suitable approach.

It is also commented “What’s the difference of organic-shelled and inorganic-shelled”, the crystal structure of DA_2_PbBr_4_ and CsPbBr_3_@Cs_4_PbBr_6_ are shown in the following (**Figure R**2). Both of them share the same functioning perovskite units, but in the former, the perovskite units are shelled by the light organic components (atomically arrayed dodecylamine ions) comprised of low-Z elements C, H and N, while in the latter they are shelled by the heavy inorganic Cs_4_PbBr_6_ composed of high-Z elements Cs, Pb and Br. Therefore, the comparison between the two systems points out the effect of light elements in enhancing the scintillation response.

**Figure R2.** Schematic views of (b) HPs with light organic shells (DA_2_PbBr_4_) and (c) HPs with inorganic heavy shells (CsPbBr_3_@Cs_4_PbBr_6_).

3. Line 231, a gradual increase in scintillation response is found as the organic cation gets bulkier. (BA is better than OA.) Therefore, what is the mainly effects between the Cs and organic? For low Z-elements and bulk volumes, which is on the dominated?

**Response:** For one thing, as we mentioned in the main manuscript that Cs^+^ builds a 3D CsPbBr_3_ perovskite structure, which is highly sensitive to charged beams, while 2D perovskites constructed by organics are much more robust. For another, the organics are composed of light elements (C, H and N), they meliorate the undesired backscattering waste of the incident β particles and suppress the energy consumption on useless signals, hence improving the capturing efficiency of β particles to enhance the scintillation response. We have introduced this content in detail in response to comment #2.

About which of low Z-elements and bulk volume dominates the scintillation response, we are not very sure which volume you mean. If you mean the volume of scintillator, one thing should be kept in mind is that β ray is different from X ray, the penetration depth of X-ray can be very strong, it requires an up to 2 mm thick MAPbBr_3_ single crystal to attenuate 98% of the photons. In contrast, the penetration power of β ray is much weaker than X-rays, a total consumption can happen within a small thickness of several tens of microns, that means the scintillation response only happens within the surface area. Therefore, volume of scintillator is not as important a factor to consider as it is in X ray or gamma ray scintillators; if you mean the volume or the 2D perovskite molecular, which gets larger as the organic cation gets bulkier with larger organic cations (butylamine, octylamine, dodecylamine, stearamine), we have explained in response to comment #1 that the organic component the incident β ray would pass through the organic layer without strong interaction, the molecular volume therefore is not a key factor.

4. The environmental stability of Cl-based 2D HPs is usually better and with low-Z element compared with I or Br. What’s the advantage of I-based and Br-based materials as β-ray scintillator?

**Response:** To realize an efficient 2D HP scintillator for β ray irradiation, a good fluorescence property is the precondition. Although Cl is lighter than Br, the pristine Cl-type 2D perovskites do not show observable fluorescence, and the situation does not get any better after doping of manganese. A comparison between the PL spectra of STA_2_PbCl_4_:Mn and STA_2_PbBr_4_:Mn is shown in **Figure R**3, the result is consistent with the conclusion in previous reports.^16, 17^

**Figure R3.** The PL spectra of STA_2_PbCl_4_:Mn and STA_2_PbBr_4_:Mn.

The problem of STA_2_PbI_4_:Mn is the insufficient exciton transfer to Mn centers. The Mn(II) center does not absorb photons itself, and relies on exciton transfer from the 2D halide perovskite host, an efficient exciton transferring to Mn centers requires a high energy offset, but STA_2_PbI_4_ does not offer large enough bandgap offset to drive the exciton transfer. Taking the CsPbX_3_ system for reference, the energy level diagram in CsPbX_3_:Mn (X=Cl, Br, and I) is shown in **Figure R**4 as follows. A strong Mn emission can only be observed in CsPbCl_3_:Mn, the exciton transfer process is gradually weakened as the Br content increases, and the Mn emission almost disappears completely in CsPbBr_3_:Mn, as presented in **Figure R**5. When the peak emission of the host reaches ~500 nm, the exciton transfer to Mn centers already becomes rather weak. In 2D system (taking STA_2_PbX_4_ for example), STA_2_PbI_4_ corresponds to CsPb(Br/I)_3_ with a ~500 nm emission peak, and the STA_2_PbBr_4_ corresponds to CsPbCl_3_ with a ~400 nm emission peak. Indeed, Mn doping does not significantly improve the emission of SA_2_PbI_4_, the comparison between the PL spectra of STA_2_PbI_4_:Mn and STA_2_PbBr_4_:Mn is shown in **Figure R**6.

**Figure R4.** The evolution of the energy level diagram of CsPbX_3_:Mn NCs during forward and reverse anion exchange. The figure is taken from “*Journal of the American Chemical Society*, *138*(45), 14954-14961” with modifications.

**Figure R5.** The evolution of CsPbCl_3−x_Br_x_:Mn NCs PL energy and intensity during anion exchange. The figure is taken from “*Journal of the American Chemical Society*, *138*(45), 14954-14961” with modifications.

**Figure R6.** PL spectra of STA_2_PbBr_4_:Mn and STA_2_PbI_4_:Mn.

Accordingly, we have added this part of analysis in the manuscript (page 10 in the revised manuscript):

“…It should be noted that 2D HPs with different halogen components were prepared, but only L_2_PbBr_4_:Mn shows respectful emitting properties, while Cl-type and I-type counterparts demonstrate rather weak fluorescence. According to previous reports, this is because L_2_PbCl_4_ are intrinsically non-emissive, and energy offset in L_2_PbI_4_ does not offer efficient exciton transfer to Mn centers.^75, 76^…”

**Corresponding Reference:**

75.	Dutta SK, Dutta A, Das Adhikari S, Pradhan N. Doping Mn^2+^ in Single-Crystalline Layered Perovskite Microcrystals. *ACS Energy Lett.* **4**, 343-351 (2019).

76.	Das Adhikari S, Guria AK, Pradhan N. Insights of Doping and the Photoluminescence Properties of Mn-Doped Perovskite Nanocrystals. *J. Phys. Chem. Lett.* **10**, 2250-2257 (2019).

5. As a β-ray scintillator detector, either the detection limitation or the pulse height spectra of isotype (like ^63^Ni@66.7keV) with the full energy peak should be given. These are two core parameters for evaluating the hard nuclear radiation detector.

**Respons**e: Unlike γ ray, the energy of β ray is continuously distributed without a characteristic peak. We then carried out the measurement of the detection limit.

The irradiation rate of was gradually reduced down to the detection limit, defined by the point where discernable scintillation disappears. Corresponding scintillation spectra and the linear response down to the limit is shown in **Figure R**7, the detection limit is measured to be 0.1 mCi, namely 3.7×10^6^ Bq. To get an idea of how small the detection limit is, we refer to the file of “*WS 533-2017 protection requirements on nuclear medicine patient*”, it states that the irradiation activity must be less than 2×10^8^ Bq (pure β ray by ^89^Sr in medicine) before the patient can be discharged, it means even in such an unoptimized measuring setup, the 2D perovskite scintillator is already ready for medical diagnosis. The irradiation activity of nuclear irradiation is much more intensive compared with radioactive medicine, the great potential of 2D perovskite scintillator for nuclear monitoring can therefore be confirmed.

**Figure R7.** (a) The scintillation spectra of the 2D HP scintillator under different β ray irradiation intensity. (b) The scintillation spectrum under the irradiation activity of 0.1 mCi. (c) The linear response of the 2D HP scintillator down to the detection limit.

Accordingly, we have added corresponding content in the manuscript as follows (page 14 in the revised manuscript):

“…Detection limit is another core parameter of scintillator detector, the measuring setup was illustrated in **Figure S**15, the irradiation activity of the β ray source was gradually reduced until a distinguishable scintillation response was not observed, the spectra are shown in Figure 5e and Figure 5f. The integration intensity of the scintillation response was linearly proportional to the irradiation activity, and the limit was determined to be 0.1 mCi (3.7×10^6^ Bq, Figure 5g). To get an idea of how small the detection limit is, we refer to the file of *“WS 533-2017 protection requirements on nuclear medicine patient”*, it states that the irradiation activity must be less than 2×10^8^ Bq (pure β ray by ^89^Sr in medicine) before the patient can be discharged, it means even in such an unoptimized measuring setup, the low-detection limit 2D HP scintillator is already ready for medical diagnosis. The irradiation activity of nuclear irradiation is much more intensive compared with radioactive medicine, the great potential of 2D HP scintillator for nuclear monitoring can therefore be confirmed…”

6. Compared with the typical scintillators such as CsI, LYSO, what is the light yield of 2D HPs scintillator?

**Response:** Since β ray can be completely absorbed within a thin surface layer, the light yield can be estimated by comparing the scintillation response with that of commercial scintillator.^18^ Here ZnS:Cu (with a high light yield towards β ray of ~100000 photons/MeV) as one of the most efficient scintillator towards β ray sensing is adopted as the reference.^19-21^ One of the most important reason for choosing ZnS:Cu is that it is in the form of powder the same as the 2D perovskite scintillator here, all the processing can be kept the same for an accurate reference. Corresponding scintillation response is shown as **Figure R**8 in the following. The integrated scintillation intensity of the STA_2_PbBr_4_:Mn scintillator is 24.7% of that of ZnS:Cu, therefore, the light yield of the 2D perovskite scintillator is estimated to be >24000 photons/MeV. This value is even higher than some typical organic scintillators including naphthalene (~15000 photons/MeV) and diphenyl (~23000 photons/MeV). Accordingly, this part has been added in the revised supplementary information file (Discussion in **Figure S**7).

**Figure R8.** Scintillation spectra of ZnS:Cu and STA_2_PbBr_4_:Mn.

Accordingly, we have added corresponding content in the manuscript (page 11 in the revised manuscript):

“…Since β ray can be completely absorbed within a thin surface layer, the light yield can be estimated by comparing the scintillation response with that of commercial scintillator, in this way, a high light yield of ~24000 photons/MeV is obtained (**Figure S**7).^36^…”

**Corresponding reference:**

36.	Mykhaylyk VB, Kraus H, Saliba M. Bright and Fast Scintillation of Organolead Perovskite MAPbBr_3_ at Low Temperatures. *Mater. Horiz.* **6**, 1740-1747 (2019).

7. The authors claim “commercial plastic scintillators is the relatively high cost”, this is also contrary to the facts. For the counter type β-ray scintillator detector, the price is mainly determined by Si-diode or PMT and readout circuit.

**Response:** According to your comment we have made clear that there is no significant cost advantage in the 2D halide perovskite scintillator compared with some commercial scintillators. To void such misleading, we have revised the content. The meaningful advantage of the 2D halide perovskite scintillator over commercial counterparts should be the facile aqueous fabrication, because the commercial plastic scintillators suffer from stringent and labour-intensive fabrication. The sentences are then revised as follows (Page 11 in the revised manuscript):

“…The commercial plastic scintillators suffer from stringent and labour-intensive fabrication. For example, the raw materials have to be highly pure, and the operations of cleaning and assembly before subsequent polymerization are detail-oriented, therefore greatly adding to the overall effort. The polymerization cycle lasts for several days, during which a persistent high-temperature treatment and a final cooling with precise control are needed to obtain stress-free products.^77, 78^…”

**Corresponding References**

77.	Pla-Dalmau A, Bross AD, Mellott KL. Low-cost extruded plastic scintillator. *Nucl. Instrum. Methods Phys. Res., Sect. A* **466**, 482-491 (2001).

78.	Zorn C. Studies in the radiation resistance of plastic scintillators: review and prospects. *Ieee. T. Nucl. Sci.* **37**, 504-512 (1990).

8. The authors claim the quite low QYs for pure 2D HPs (<8%), but the Mn(II) doped BA_2_PbBr_4_, OA_2_PbBr_4_, DA_2_PbBr_4_, STA_2_PbBr_4_ were all significantly enhanced. However, the radius of Mn is less than Pb and there is a large difference in atom size, which affects the crystal structure. What about other divalent cation? What’s the doping concentration in these samples?

**Response:** Indeed, the radius of Mn(II) in an octahedral coordination 0.97 Å, which is smaller than that of Pb^2+^ (1.33 Å) in halide perovskites,^22^ but the doping level is so low (~0.3%, measured by ICP-MS) that the crystal structure will hardly be influenced. Corresponding powder X-ray diffraction patterns of BA_2_PbBr_4_, OA_2_PbBr_4_, DA_2_PbBr_4_, STA_2_PbBr_4_ before and after the doping procedure were provided in the supporting information (**Figure S**4 in the previous manuscript, **Figure S**5 in the revised manuscript), and are showcased in the following (**Figure R**9). It should be noted that owing to the 2D morphology, the 2D perovskites easily lie flat to the substrate and therefore present clear orientation during XRD characterization to present mainly (00*l*) facets. But the doping of Mn^2+^ involves mechanical grinding, which breaks the previous 2D morphology to present more detailed X-ray diffraction features. Indeed, the characteristic diffraction peaks are all well-remained after the Mn doping procedure, which powerfully negates the possibility of structure decomposition after Mn doping. It should also be noted that Mn(II) has already been demonstrated as an effective dopant in 2D HP systems in the literature.^23^

**Figure R9.** XRD patterns of a series of pristine and Mn(II)-doped 2D perovskites. Corresponding organic components are (a) butylamine (BA), (b) octylamine (OA), (c) dodecylamine (DA), (d) stearamine, respectively.

As for the other divalent ions, typical divalent ions including Mg^2+^,^24^ Ca^2+^,^25^ Sr^2+^,^26^ Ba^2+^,^27^ Ni^2+^,^28^ Co^2+^,^29^ Cu^2+^,^30^ Zn^2+^,^31^ Sn^2+^,^32^ Fe^2+^ and so on,^33^ have been extensively studied as dopants in HP systems, they have been observed to influence the crystallization process, bandgap or stability, but not to serve as emitting centers to enhance the overall emission the same as Mn^2+^. The fascinating feature of Mn^2+^ is that it “recycles” the wasted energy from mid-trap states rather than competes with the band-edge emission of the host, hence transforming the non-radiative waste into radiative recombination to improve the scintillation response. More importantly, β ray absorption happens within a shallow surface of scintillators in the front, the detector is equipped on the rear, the produced visible light has to pass through the scintillator to be collected. Mn centers produce a down-converted light compared with the host, so that the scintillation response can be effectively sensed without self-absorption loss.

To measure the doping concentration of Mn (II), ICP-MS (Agilent 7700) was carried out (we did the test before and described the way of measurement in the Method section, but forgot to provide the data, sorry for the carelessness). The nominal doping concentration was 10%, but the real doping concentration measured by the ICP-MS is only 0.26%~0.31% as shown in the following:

**Table R1.** The nominal doping concentration of Mn.

| Material | Doping concentration |
| --- | --- |
| BA_2_PbBr_4_:Mn | 0.29% |
| OA_2_PbBr_4_:Mn | 0.26% |
| DA2PbBr4:Mn | 0.31% |
| STA_2_PbBr_4_:Mn | 0.27% |

Accordingly, we have added corresponding contents in the revised manuscript as follows (page 10 in the revised manuscript), and the above **Table R**1 has been added as **Table S**1 in the supporting information file:

“…The doping concentration was measured to be 0.26%~0.31% (**Table S**1) by inductively coupled plasma mass spectrometry (ICP-MS), the concentration is so low that even though the ion radius of Mn^2+^ (0.97 Å) is much smaller than Pb^2+^ (1.33 Å), the integrity of 2D HP lattice structure will hardly be compromised, as is also confirmed by the XRD patterns in **Figure S**5…”

9. The peak positions for both undoped sample (shown in Fig. S2) and doped samples are all in the same wavelength ~610 nm. As the authors mentioned the trap states of Mn(II) were response for the enhanced luminescence, the position should change before and after doping. In fact, the luminescences come from the self-trap states from the PbI_6_ octahedron is usually found in low dimensional halide perovskites. Therefore, the corresponding discussion is misleading. Besides, the intensity of luminescence is also influenced by the crystalline quality of samples, from the pictures in Fig. 3(a)-(d), however, which is ignored by the authors.

**Response:** It seems there is a misunderstanding. It should be made clear the difference between the spectra of pure 2D perovskites and Mn-doped 2D perovskites, as shown in **Figure R**10 in the following. The intrinsic emission of pure 2D HP is peaked at ~400 nm, and the emission from Mn centers is at ~610 nm, they are easy to distinguish. The spectra in **Figure S**2 refers to that of DA_2_PbBr_4_:Mn rather than pure DA_2_PbBr_4_, the reason that Mn doping is not mentioned in the **Figure S**2 part is because we meant to arrange the systematic introduction of Mn doping in the following text, while the **Figure S**2 part lays emphasis on the importance of the light organic component. In order to prevent such misleading, we have added corresponding description in the manuscript (Page 8 in the revised manuscript)

“…Here DA_2_PbBr_4_ (C_24_H_54_N_2_PbBr_4_, DA is dodecylamine) is selected as the representative 2D HP, a doping strategy was applied to improve the PL QY of the 2D HP as will be introduced in the following...”

The Figure caption of **Figure S**2 in the supporting information has also been revised as:

“…**Figure S2.** Scintillation response of inorganic-shelled and organic shelled HPs towards β ray. The 2D system refers to DA_2_PbBr_4_:Mn (DA is dodecylamine)…”

**Figure R10.** The PL spectra of pure SA_2_PbBr_4_ and SA_2_PbBr_4_:Mn.

As well, the self-trapped emission in 2D perovskites is largely different from the that of Mn-doped 2D perovskites. On the one hand, the former features a broadband emission to even cover the whole visible spectrum in particular systems,^34-37^ while the fluorescence spectrum of the latter is composed of two distinguishable peaks at ~400 nm (host) and ~610 nm (Mn centers), respectively; on the other hand, the broadband self-trapped emission is from the intrinsic self-trapped excitons, which already exist in pure 2D perovskites but do not require functional dopants. Indeed, we do not observe such a broadband emission in pure 2D perovskites here (as can be checked in **Figure R**10).

It was also commented that we should notice that the crystallinity might influence the emission intensity of the samples. The fact is that when Mn (II) dopants are incorporated, a grinding procedure and a subsequent annealing treatment were carried out as we have introduced in the Method section. Figure 3(a)-(d) in the manuscript showcase the pristine 2D perovskites with different organic cations, the scanning electron microscopy (SEM) images of them after the grinding procedure are shown in the following (**Figure R**11). Clearly, a ripening effect takes place in the 2D halide perovskites after the grinding and annealing procedures, and no a crystallinity difference will be identified between them. The annealing treatment at 100 degrees Celsius will not cause the loss of organics in 2D perovskites, which only happens at a temperature above 300 degrees Celsius according to the thermogravimetric analysis.

**Figure R11**. SEM images of BA_2_PbBr_4_:Mn, OA_2_PbBr_4_:Mn, DA_2_PbBr_4_:Mn and STA_2_PbBr_4_:Mn, respectively, after the Mn doping procedures, including grinding and annealing (100 degrees Celsius for 20 mins in nitrogen). The scale bars are 10 μm.

Accordingly, we have added this part in the revised manuscript (page 10 in the revised manuscript):

“…The doping procedure includes grinding and thermal treatment, which leads to ripening of the 2D HPs (**Figure S**4)…”

10. How the hydrophobicity of organic cations in 2D HPs determine whether it can be synthesized by water?

**Response:** For all the three types of 3D halide perovskites, including CsPbX_3_, CH_3_NH_3_PbX_3_ and CH(NH)NH_3_PbX_3_ all decompose into PbX_2_ upon water treatment. Taking CH_3_NH_3_PbBr_3_ for example, when touching water, CH_3_NH_3_Br will be dissolved out and leaves behind PbBr_2_, this is because CH_3_NH_3_Br is hydrophilic, while PbBr_2_ is poorly soluble in water.

2D halide perovskites (n=1 here) follow a chemical formula of L_2_PbBr_4_, L is the organic cation, they can be obtained by combining 2:1 molar ratio of LBr and PbBr_2_. Therefore, if LBr is water-insoluble the same as PbBr_2_, then pure water can be used as the antisolvent to force the cocrystallization of LBr and PbBr_2_ to form L_2_PbBr_4_. It should be noted that the extensively studied 2D perovskite systems, such as phenylethylamine (PEA)-type and butyl amine (BA)-type 2D perovskites, fail the aqueous synthesis owing to the hydrophilicity of PEA and BA.^38, 39^ But dodecylamine (DA) and stearamine (SA) are intrinsically water-stable, therefore empowering the 2D perovskites with superior water robustness and aqueous synthesis.

11. There is only one peak in Fig. 4(c) the PL spectra, which is not consistent with the pure 2D halide perovskites shown in the manuscripts (Fig. S2).

**Response:** We have explained in the above (response to comment #9) that **Figure S**2 shows the PL spectrum of Mn(II)-doped 2D perovskite (DA_2_PbBr_4_:Mn), while Figure 4c presents a series of pristine 2D halide perovskites without Mn(II) dopants. Therefore, in **Figure S**2 there will be an additional Mn(II) peak at ~610 nm.

12. In terms of thermotolerance, however, the stability of phase or composition can’t totally represent the working stability in high temperature. The performance of the scintillators in high temperature under the illumination of β-ray is critically required, for example, light yield, decay time, and so on. The data in Fig. 5(b) looks beautiful, but is quite limited and the accuracy of ordinate is very poor.

**Response:** We have according to your comment conducted supplementary characterizations to give an overall high-temperature performance. The STA_2_PbBr_4_:Mn was heated under various temperatures, and the fluorescence spectra were measured in situ using MAX2000-Pro fiber optic spectrometer. The temperature PL spectra are shown in **Figure R**12 in the following:

**Figure R12**. Temperature-dependent PL spectra of STA_2_PbBr_4_:Mn.

The result shows that even when the temperature reaches beyond 110 degrees Celsius, at which point commercial plastic scintillators already fail owing to a glass transition of the bases (<105 degrees Celsius), STA_2_PbBr_4_:Mn still retains 34% of the overall integrated PL intensity, which corresponds to a light yield of approximately 8100 photons/MeV.

As for the decay time, we are sorry that we cannot find a lab to support the temperature-controlled PL lifetime measurement during this special period, hope you understand that. However, we have referenced the literature on CaAl_4_O_7_:Mn and CsPbCl_3_:Mn, ^40, 41^ corresponding temperature-dependent PL decays are shown in **Figure R**13 in the following, it shows that the decay time decreases as the temperature increases, which is owing to an accelerated quenching process at elevated temperatures, and the faster decay rate is desirable for the scintillator.

**Figure R13.** (a) Temperature-dependent time-resolved Mn emission lifetime of CaAl_4_O_7_:Mn, the figure is reproduced from ref. 41.^40^ (b) Temperature-dependent time-resolved Mn emission lifetime of CsPbCl_3_:Mn, the figure is reproduced from ref. 42.^41^

Accordingly, we have added the following content in the manuscript (page 12 in the revised manuscript):

“…The *in situ* fluorescence at elevated temperatures was also measured (**Figure S**11), the result shows that even when the temperature reaches beyond 110 degrees Celsius, at which point commercial plastic scintillators already fail owing to a glass transition of the bases (<105 degrees Celsius), STA_2_PbBr_4_:Mn still retains 34% of the overall integrated PL intensity, which corresponds to a light yield of approximately 8100 photons/MeV…”

It is also commented that the ordinate accuracy of Fig. 5(b) is very poor. We guess it is in partial due to the resolution reduction after being processed by the submitting system, and we have adjusted the figure as shown in the following (**Figure R**14):

**Figure R14**. **Characterizations of thermotolerance and irradiation stability of the 2D HP-based β ray scintillators for imaging.** (a) TGA characterization of the 2D perovskites. The decomposition temperature is approximately 300 degrees Celsius. (b) Evolution of integrated scintillation intensity of the Mn-doped 2D perovskites during long-time radiation test. Inset: schematic view of the scintillation behaviour of the Mn-doped 2D perovskites. (c) Illustration of the setup of the imaging system. (d) The obtained imaging patterns. (e) The scintillation spectra of the 2D HP scintillator under different β ray irradiation intensity. (f) The scintillation spectrum under the irradiation activity of 0.1 mCi. (g) The linear response of the 2D HP scintillator down to the detection limit.

13. If I understand the result in Fig. 5(c) correctly, the imaging shows the area with 2D HPs scintillator, which do not help for the real application. What’s the space resolution?

**Response:** Yes, we have according to your comment made clear the result in essence shows that the 2D perovskite scintillator enables effective and highly discernable response to β ray, rather than imaging in the traditional sense. Accordingly, we have changed the manuscript to as follows (Page 14 in the revised manuscript)

“…Proof-of-concept radiation test is further demonstrated to show that the 2D HP scintillator offers effective and highly discernable response to β ray. The set-up of a self-built measurement system is shown in Figure 5c. The incident β ray is firstly shaped by masks of a series of hollowed-out letters “NJ”, “JU”, “US”, “ST” (**Figure S**13) before working on the 2D HP active layer, the produced scintillation is then captured by an EMCCD camera. Clearly, the obtained patterns perfectly reproduce these letters in masks (Figure 5d), while there is no fluorescence signal in the nonresponsive region…”

14. The afterglow effect is very important for imaging using scintillator detector. The 2D HPs result should compare with the typical scintillators, like LYSO, CsI. Besides, the time response (resolution) should be added in the manuscript.

**Response:** In fact, we have provided the analysis of afterglow of the 2D perovskite scintillator in the previous manuscript. The afterglow curves were measured by using a pulse 375 nm excitation (pulse duration of 50 ps, both β ray and ultraviolet light have a small penetration depth, therefore their afterglow should be the same), the afterglow was recorded in oscilloscope mode using a SPCM-AQRH-15 APD detector. The afterglow level was calculated as a ratio between the luminescence intensities (I_0_) measured during the X-ray irradiation pulse and at the time delay t after the irradiation is terminated, namely η(t) = I(t)/I_0_ × 100%. The 2D perovskite scintillator was placed in total darkness for 24 h before the afterglow measurement was carried out.

In general, to obtain a high-contrast imaging without lag, the signal has to be attenuated by at least three orders of magnitude in several milliseconds after irradiation cutoff.^42^ As shown in **Figure R**15, the afterglow at 5 μs, 6 μs and 7 μs are 5.77‰, 2.18‰ and 1.26‰, respectively, which indicates a low afterglow effect in the 2D perovskite scintillator. In contrast, it requires 80000 s to decay to 3‰ afterglow for LYSO scintillator,^43^ and Cs:Tl needs 2 ms to attenuate the scintillation to 2‰ (both towards X ray rather than β ray).^44^ It is noteworthy that the decay of many scintillators is composed of several fast and slow components, the fast component determines the general decay time of scintillator, but the slow decay may still cause a considerable afterglow scintillation after a long time of shutting off the excitation. However the scintillation of the 2D perovskite scintillator here is dominated by the Mn centers with single recombination path, therefore the decay almost follows a standard monoexponential fit.

**Figure R15.** Afterglow of STA_2_PbBr_4_:Mn after cutoff of excitation.

Another adamant proof of the weak afterglow is the steady scintillation response after ling-time irradiation by β ray. That is, if there is a strong afterglow of the 2D perovskite scintillator, the scintillation response should be enhanced after the long-time irradiation, but the scintillation response remains quite stable even after 15 h β ray irradiation at a dose rate of 0.67 kGy/h (as will be soon introduced in the following response).

The decay time data of 0.5 ms is also added in the revised manuscript. Accordingly, we have added the following description (page 13 in the revised manuscript):

“…As shown in **Figure S**14, the decay time is 0.5 ms, and the relative afterglow intensities at 5 μs, 6 μs and 7 μs are 5.77‰, 2.18‰ and 1.26‰, while for comparison the characteristic afterglows of CsI:Tl and LYSO for X-ray reach several microseconds and several hours, respectively.^89, 90^…”

**Corresponding reference:**

89.	Brecher C, et al. Suppression of afterglow in CsI:Tl by codoping with Eu^2+^—I: Experimental. Nucl. Instrum. Methods Phys. Res., Sect. A 558, 450-457 (2006).

90.	Mao R, Wu C, Dai LE, Lu S. Crystal growth and scintillation properties of LSO and LYSO crystals. J. Cryst. Growth 368, 97-100 (2013).

15. In Fig. S9, the authors show the comparisons of the PL spectra before and after the radiation tests, however, the curves have very week SN ratio, which is insufficient for claim the sample with superior irradiation hardness.

**Response:** The seemingly weak signal-to-noise ratio does not mean that the radiation intensity is weak, the irradiation rate is surest, the main reason is the measuring setup. Because it is a long-time test (lasting for 15 h), the measurement setup is different from typical scintillation response test, so as to avoid the damage caused by the β ray irradiation. The schematic illustration is shown in the following (**Figure R**16), for the 2D halide perovskite scintillator, the incident β ray was perpendicular to the outgoing light path, the distance between the detector to read out the scintillation spectrum and the scintillator is set to be much larger than that of typical scintillation response test. Such setup also increases the interaction between β ray source and the crate, which is made of eupolymer, to produce a strong noise background.

**Figure R16.** Schematic illustration of the scintillation measuring setup.

The seemingly weak S/N ratio is also partially due to the overlapping of the three curves of spectrum, if it was plotted in the waterfall form, the signal/noise ratio looks much better (**Figure R**17):

**Figure R17.** The evolution of scintillation spectra of STA_2_PbBr_4_:Mn during the irradiation test.

Anyway, the irradiation rate and the accumulated irradiation dose are surest, the seemingly large S/N ratio is not owing to an insufficient irradiation but a measuring setup.

At last, we would like to thank you again for your time and valuable comments. We sincerely hope the point-to-point reply fully addresses your concerns. Any more comments to strengthen the manuscript are highly welcomed, thanks a lot.

**Response to Reviewer #2**

This paper presents a breakthrough in my mind. Perovskites for beta-ray scintillation is a totally new idea. The materials tested are very well selected and designed for optimized scintillation. The stability of the perovskites in water is impressive. The only important parameter I miss in this paper is the detection limit, which should be provided in Bq/min and added for the various materials tested.

**Response:** Dear Reviewer, thank you so much for your time and the valuable comments, and we have carefully revised the manuscript accordingly to enhance the manuscript, we hope the new version of manuscript addresses your concern. All the revisions in the manuscript and in this reply file have been highlighted in yellow.

We carried out the measurement of the detection limit. It should be noted that the detection limit of a scintillator detector is not an absolute value, it is highly related to the matching degree of fluorescence wavelength and photomultiplier tube, and is also determined by the exposure time, the longer the exposure time, the lower detection limit. Therefore, the detection limit is only meaningful when compared with reference scintillators. Since the emission peak of most commercial scintillator plastic for β ray detection is within the ultraviolet and blue light range, which is quite different from the 2D perovskite scintillator (~610 nm) in this work, therefore, ZnS:Cu with an emission peak of 530 nm is used as the reference scintillator.

One thing we should know about ZnS:Cu is that is offers a quite high physical light yield (~100000 photons/MeV), i.e., the fraction of the absorbed radiation energy transformed into light, but the practical light yield, defined as the amount of light obtainable from a given intensity of radiation, is greatly compromised by the strong self-absorption, because in a detector device the scintillation is produced in the front of scintillator and has to pass through it to reach the PMT in the rear.^19^ In this regard the measuring setup was designed as shown in **Figure R**18, the ZnS:Cu was excited by β ray to produce scintillation that was directly measured without passing through the ZnS:Cu scintillator, the detection limit of 2D perovskite scintillator was measured the same way for comparison.

**Figure R18.** The measuring setup for the detection limit.

The irradiation rate of was gradually reduced down to the detection limit, defined by the point where discernable scintillation disappears. Corresponding scintillation spectra and the linear response down to the limit is shown in **Figure R**19, the detection limit is measured to be 0.1 mCi, namely 3.7×10^6^ Bq, comparable to that of ZnS:Cu (1.5×10^6^ Bq). To get an idea of how small the detection limit is, we refer to the file of “*WS 533-2017 protection requirements on nuclear medicine patient*”, it states that the irradiation activity must be less than 2×10^8^ Bq (pure β ray by ^89^Sr in medicine) before the patient can be discharged, it means even in such an unoptimized measuring setup, the 2D perovskite scintillator is already ready for medical diagnosis. The irradiation activity of nuclear irradiation is much more intensive compared with radioactive medicine, the great potential of 2D perovskite scintillator for nuclear monitoring can therefore be confirmed.

**Figure R19.** (a) The scintillation spectra of the 2D HP scintillator under different β ray irradiation intensity. (b) The scintillation spectrum under the irradiation activity of 0.1 mCi. (c) The linear response of the 2D HP scintillator down to the detection limit.

Accordingly, we have added corresponding content in the manuscript as follows (page 14 in the revised manuscript), and the details abovementioned are added in the Discussion part in **Figure S**15 in the supplementary file:

“…Detection limit is another core parameter of scintillator detector, the measuring setup was illustrated in **Figure S**15, the irradiation activity of the β ray source was gradually reduced until a distinguishable scintillation response was not observed, the spectra are shown in Figure 5e and Figure 5f. The integration intensity of the scintillation response was linearly proportional to the irradiation activity, and the limit was determined to be 0.1 mCi (3.7×10^6^ Bq). To get an idea of how small the detection limit is, we refer to the file of *“WS 533-2017 protection requirements on nuclear medicine patient”*, it states that the irradiation activity must be less than 2×10^8^ Bq (pure β ray by ^89^Sr in medicine) before the patient can be discharged, it means even in such an unoptimized measuring setup, the low-detection limit 2D perovskite scintillator is already ready for medical diagnosis. The irradiation activity of nuclear irradiation is much more intensive compared with radioactive medicine, the great potential of 2D perovskite scintillator for nuclear monitoring can therefore be confirmed.…”

At last, we would like to thank you again for your time and valuable comments. We sincerely hope the point-to-point reply fully addresses your concern. Any more comments to strengthen the manuscript are highly welcomed, thanks a lot.

**References**

1.	Yakunin S*, et al.* Detection of gamma photons using solution-grown single crystals of hybrid lead halide perovskites. *Nature Photonics* **10**, 585 (2016).

2.	Wei H*, et al.* Dopant compensation in alloyed CH_3_NH_3_PbBr_3-x_Cl_x_ perovskite single crystals for gamma-ray spectroscopy. *Nat. Mater.* **16**, 826-833 (2017).

3.	Nazarenko O, Yakunin S, Morad V, Cherniukh I, Kovalenko MV. Single crystals of caesium formamidinium lead halide perovskites: solution growth and gamma dosimetry. *NPG Asia Mater.* **9**, e373 (2017).

4.	He Y*, et al.* High spectral resolution of gamma-rays at room temperature by perovskite CsPbBr_3_ single crystals. *Nat. Commun.* **9**, 1609 (2018).

5.	Yakunin S*, et al.* Detection of X-ray photons by solution-processed lead halide perovskites. *Nat. Photonics* **9**, 444 (2015).

6.	Wei H*, et al.* Sensitive X-ray detectors made of methylammonium lead tribromide perovskite single crystals. *Nat. Photonics* **10**, 333 (2016).

7.	Heiss W, Brabec C. Perovskites target X-ray detection. *Nat. Photonics* **10**, 288-289 (2016).

8.	Birowosuto MD*, et al.* X-ray Scintillation in Lead Halide Perovskite Crystals. *Sci. Rep.* **6**, 37254 (2016).

9.	Lang F*, et al.* Radiation Hardness and Self-Healing of Perovskite Solar Cells. *Adv. Mater.* **28**, 8726-8731 (2016).

10.	Cardinaletti I*, et al.* Organic and perovskite solar cells for space applications. *Sol. Energ. Mat. Sol. C.* **182**, 121-127 (2018).

11.	Miyazawa Y*, et al.* Tolerance of Perovskite Solar Cell to High-Energy Particle Irradiations in Space Environment. *iScience* **2**, 148-155 (2018).

12.	Wei H, Huang J. Halide Lead Perovskites for Ionizing Radiation Detection. *Nat. Commun.* **10**, 1066 (2019).

13.	Paternò G*, et al.* Perovskite solar cell resilience to fast neutrons. *Sustainable Energy & Fuels*, (2019).

14.	Tu Y*, et al.* Mixed-cation perovskite solar cells in space. *Sci. China: Phys., Mech. Astron.* **62**, 974221 (2019).

15.	Kanaya S*, et al.* Proton Irradiation Tolerance of High Efficiency Perovskite Absorbers for Space Applications. *J. Phys. Chem. Lett.*, (2019).

16.	Das Adhikari S, Guria AK, Pradhan N. Insights of Doping and the Photoluminescence Properties of Mn-Doped Perovskite Nanocrystals. *J. Phys. Chem. Lett.* **10**, 2250-2257 (2019).

17.	Dutta SK, Dutta A, Das Adhikari S, Pradhan N. Doping Mn^2+^ in Single-Crystalline Layered Perovskite Microcrystals. *ACS Energy Lett.* **4**, 343-351 (2019).

18.	Mykhaylyk VB, Kraus H, Saliba M. Bright and Fast Scintillation of Organolead Perovskite MAPbBr_3_ at Low Temperatures. *Mater. Horiz.* **6**, 1740-1747 (2019).

19.	Kallmann H. Quantitative Measurements with Scintillation Counters. *Phys. Rev.* **75**, 623-626 (1949).

20.	Xu Z-H, Tang X-B, Hong L, Liu Y-P, Chen D. Structural effects of ZnS:Cu phosphor layers on beta radioluminescence nuclear battery. *J. Radioanal. Nucl. Ch.* **303**, 2313-2320 (2014).

21.	Russo J, Litz M, Ray W, 2nd, Smith B, Moyers R. A radioluminescent nuclear battery using volumetric configuration: (63)Ni solution/ZnS:Cu,Al/InGaP. *Appl. Radiat. Isot.* **130**, 66-74 (2017).

22.	Mir WJ, Jagadeeswararao M, Das S, Nag A. Colloidal Mn-doped cesium lead halide perovskite nanoplatelets. *ACS Energy Lett.* **2**, 537-543 (2017).

23.	Biswas A, Bakthavatsalam R, Kundu J. Efficient Exciton to Dopant Energy Transfer in Mn^2+^-Doped (C_4_H_9_NH_3_)_2_PbBr_4_ Two-Dimensional (2D) Layered Perovskites. *Chem. Mater.* **29**, 7816-7825 (2017).

24.	Yang F*, et al.* Magnesium-Doped MAPbI3 Perovskite Layers for Enhanced Photovoltaic Performance in Humid Air Atmosphere. *ACS Appl Mater Interfaces* **10**, 24543-24548 (2018).

25.	Lu C*, et al.* Calcium doped MAPbI_3_ with better energy state alignment in perovskite solar cells. *Appl. Phys. Lett.* **112**, 193901 (2018).

26.	Lau CFJ*, et al.* Strontium-doped low-temperature-processed CsPbI_2_Br perovskite solar cells. *ACS Energy Lett.* **2**, 2319-2325 (2017).

27.	Chan S-H, Wu M-C, Lee K-M, Chen W-C, Lin T-H, Su W-F. Enhancing perovskite solar cell performance and stability by doping barium in methylammonium lead halide. *J. Mater. Chem. A* **5**, 18044-18052 (2017).

28.	Yong ZJ*, et al.* Doping-Enhanced Short-Range Order of Perovskite Nanocrystals for Near-Unity Violet Luminescence Quantum Yield. *J. Am. Chem. Soc.* **140**, 9942-9951 (2018).

29.	Klug MT*, et al.* Tailoring metal halide perovskites through metal substitution: influence on photovoltaic and material properties. **10**, 236-246 (2017).

30.	Bi C, Wang S, Li Q, Kershaw SV, Tian J, Rogach AL. Thermally Stable Copper(II)-Doped Cesium Lead Halide Perovskite Quantum Dots with Strong Blue Emission. *J. Phys. Chem. Lett.* **10**, 943-952 (2019).

31.	Zhao W, Yang D, Yang Z, Liu S. Zn-doping for reduced hysteresis and improved performance of methylammonium lead iodide perovskite hybrid solar cells. *Mater. Today Energy* **5**, 205-213 (2017).

32.	Hao F, Stoumpos CC, Chang RP, Kanatzidis MG. Anomalous band gap behavior in mixed Sn and Pb perovskites enables broadening of absorption spectrum in solar cells. *J. Am. Chem. Soc.* **136**, 8094-8099 (2014).

33.	Poindexter JR*, et al.* Distribution and Charge State of Iron Impurities in Intentionally Contaminated Lead Halide Perovskites. *IEEE Journal of Photovoltaics* **8**, 156-161 (2017).

34.	Dohner ER, Jaffe A, Bradshaw LR, Karunadasa HI. Intrinsic White-Light Emission from Layered Hybrid Perovskites. *J. Am. Chem. Soc.* **136**, 13154-13157 (2014).

35.	Dohner ER, Hoke ET, Karunadasa HI. Self-assembly of broadband white-light emitters. *J. Am. Chem. Soc.* **136**, 1718-1721 (2014).

36.	Thirumal K*, et al.* Morphology-Independent Stable White-Light Emission from Self-Assembled Two-Dimensional Perovskites Driven by Strong Exciton–Phonon Coupling to the Organic Framework. *Chem. Mater.* **29**, 3947-3953 (2017).

37.	Wang S*, et al.* Highly efficient white-light emission in a polar two-dimensional hybrid perovskite. *Chem Commun (Camb)* **54**, 4053-4056 (2018).

38.	Ma C, Lo MF, Lee CS. Stabilization of organometallic halide perovskite nanocrystals in aqueous solutions and their applications in copper ion detection. *Chem Commun (Camb)* **54**, 5784-5787 (2018).

39.	Passarelli JV*, et al.* Enhanced Out-of-Plane Conductivity and Photovoltaic Performance in n = 1 Layered Perovskites through Organic Cation Design. *J. Am. Chem. Soc.* **140**, 7313-7323 (2018).

40.	Li P*, et al.* Temperature dependent red luminescence from a distorted Mn^4+^ site in CaAl_4_O_7_:Mn^4+^. *Opt. Express* **21**, 18943-18948 (2013).

41.	Wu W, Liu W, Wang Q, Han Q, Yang Q. Temperature-dependent photoluminescence of pure and Mn-doped CsPbCl3 nanocrystals. *J. Alloy. Compd.* **787**, 165-172 (2019).

42.	Yang B*, et al.* Lead-Free Halide Rb_2_CuBr_3_ as Sensitive X-Ray Scintillator. *Adv. Mater.* **31**, e1904711 (2019).

43.	Mao R, Wu C, Dai LE, Lu S. Crystal growth and scintillation properties of LSO and LYSO crystals. *J. Cryst. Growth* **368**, 97-100 (2013).

44.	Brecher C*, et al.* Suppression of afterglow in CsI:Tl by codoping with Eu^2+^—I: Experimental. *Nucl. Instrum. Methods Phys. Res., Sect. A* **558**, 450-457 (2006).

**Reviewer comments, second version:**

REVIEWERS' COMMENTS:

Reviewer #1 (Remarks to the Author):

This paper offers the readers with sufficient information to learn about β ray sensing. Besides, I think this work will be a good supplement to the extensively studied X-ray and γ ray detection based on halide perovskite materials. I suggest the publication and the following considerations are suggested for authors before publication:

1. The aqueous synthesis is scalable, and also offers tunability over emission by varying the halogen component, the authors claimed it means good potential for designing emission-tunable scintillator for β ray. However, except Br- and I- perovskites, the others seem weak in emission? Or should the doping strategy also enhance the emission?
2. In Table 1, the ceiling point is actually the decomposition temperature, it can be meaningless because before reaching the decomposition temperature the scintillators have already failed owing to thermal quenching, probably 110 degrees Celsius for the two-dimensional halide perovskites?
3. I also go through the details, the authors should be very careful about the details in the Method part. For example, the Mn salt is not introduced in the Chemicals; PbI2 not PbBr2 is used when preparing BA2PbBr4 and OA2PbBr4? The absorption measurement and contact angle test are introduced in Characterizations, but the data are missing in the manuscript.


Reviewer #2 (Remarks to the Author):

The authors have perfectly reacted to the reviewers comments and added the suggested missing data. Thus, I suggest to publish as is.

**Author rebuttal, second version:**

**Point-to-point Reply to Referees**

**Reviewer#1: This paper offers the readers with sufficient information to learn about β ray sensing. Besides, I think this work will be a good supplement to the extensively studied X-ray and γ ray detection based on halide perovskite materials. I suggest the publication and the following considerations are suggested for authors before publication:**

**Response:** Thank you very much for your time and valuable comments, according to which we have revised and enhanced the manuscript. We sincerely hope the following revisions fully address your concerns.

**1. The aqueous synthesis is scalable, and also offers tunability over emission by varying the halogen component, the authors claimed it means good potential for designing emission-tunable scintillator for β ray. However, except Br- and I- perovskites, the others seem weak in emission? Or should the doping strategy also enhance the emission?**

**Response:** If the emission intensity is taken into consideration, the statement may not be accurate enough, and we have removed it in the updated manuscript. Our considerations are as follows:

The halogen tunability of the 2D halide perovskite products is meant to show the universality of the aqueous synthesis, and the 2D halide perovskites indeed show halogen-dependent band-edge emission.

But it is true that all the phase-pure 2D halide perovskites, even including Br-type and I-type ones, are weak in emission. That is why Mn doping strategy is adopted, aiming to improve the emission of the Br-type 2D halide perovskite for β ray scintillation. According to literature, the weak emission may be ascribed to the intrinsic strong electron-phonon interaction.^[1]^

Doping has been proved to be an efficient strategy to improve the emission intensity of 2D halide perovskites, but then the emission mainly comes from the doping center in most cases rather than the band-edge recombination, and it would no longer be “emission tunable” scintillators.

Thank you very much for the reminder.

**2. In Table 1, the ceiling point is actually the decomposition temperature, it can be meaningless because before reaching the decomposition temperature the scintillators have already failed owing to thermal quenching, probably 110 degrees Celsius for the two-dimensional halide perovskites?**

**Response:** Indeed, the ceiling point refers to the decomposition temperature. About the temperature-dependent scintillation behavior, we have introduced in the main text in detail, clearly showing that the 2D halide perovskite scintillator is much more robust compared to commercial counterparts when heated. The 2D halide perovskite scintillator maintains 34% overall integrated emission intensity even at 110 degrees Celsius, at which point the commercial plastic scintillators already fail owing to glass transition.

However, such details cannot be summarized in table, and **to offer an intuitive impression for readers, the decomposition temperature (the so-called “ceiling point”) is listed for comparison.**

We have modified the “ceiling point” into “decomposition point” for accuracy in the updated manuscript, thank you for this comment.

**3. I also go through the details, the authors should be very careful about the details in the Method part. For example, the Mn salt is not introduced in the Chemicals; PbI_2_ not PbBr_2_ is used when preparing BA_2_PbBr_4_ and OA_2_PbBr_4_? The absorption measurement and contact angle test are introduced in Characterizations, but the data are missing in the manuscript.**

**Response:** We have gone through the manuscript and supplementary file very carefully to fix these careless mistakes, the traces can be found in manuscript word file.

At last, we would like to thank you again for your time and valuable comments. We sincerely hope the revised manuscript fully addresses your concerns, and meets the requirements of ***Nature Communications***.

**Reviewer#2: The authors have perfectly reacted to the reviewers comments and added the suggested missing data. Thus, I suggest to publish as is.**

**Response:** Thank you very much for your time and positive comments. We sincerely hope the updated manuscript meets the requirements of ***Nature Communications***.

**Reference**

[1] X. Gong, O. Voznyy, A. Jain, et al. Electron–phonon interaction in efficient perovskite blue emitters[J]. Nature Materials, 2018, 17(6): 550-556

**Reviewer comments, third version:**

**Author rebuttal, third version:**
